# Coupled Domain-Boundary Variational Formulations for Hodge–Helmholtz Operators

**DOI:** 10.1007/s00020-022-02684-6

**Published:** 2022-02-03

**Authors:** Erick Schulz, Ralf Hiptmair

**Affiliations:** ETH Zürich, SAM, HG G 58.3, 8092 Zürich, Switzerland

**Keywords:** Hodge–Laplace equation, Hodge–Helmholtz equation, Hodge decomposition, Calderón projector, Symmetric coupling, T-coercivity, 35Q61, 35Q60, 65N30, 65N38, 78A45, 78M10, 78M15

## Abstract

We couple the mixed variational problem for the generalized Hodge–Helmholtz or Hodge–Laplace equation posed on a bounded 3D Lipschitz domain with the first-kind boundary integral equations arising from the latter when constant coefficients are assumed in the unbounded complement. Recently developed Calderón projectors for the relevant boundary integral operators are used to perform a symmetric coupling. We prove stability of the coupled problem away from resonant frequencies by establishing a generalized Gårding inequality (T-coercivity). The resulting system of equations describes the scattering of monochromatic electromagnetic waves at a bounded inhomogeneous isotropic body possibly having a “rough” surface. The low-frequency robustness of the potential formulation of Maxwell’s equations makes this model a promising starting point for Galerkin discretization.

## Introduction

Let $\Omega _s\subset {\mathbb {R}}^3$ be a bounded Lipschitz domain [32, Def. 2.1] representing a region of space occupied by a dielectric object, the scatterer, with spatially varying material properties. The scalar material coefficients are assumed to be bounded, i.e. $\mu ,\epsilon \in L^{\infty }({\mathbb {R}}^3)$. In a non-dissipative medium, the functions $\mu$ and $\epsilon$ are real-valued and uniformly positive. Dissipative effects are captured by allowing the coefficients to have non-negative imaginary parts [5, Sec. 1.1.3]. We follow [21] and suppose that

$$\begin{aligned}&0<\mu _{\text {min}}\le \mathfrak {Re}(\mu )\le \mu _{\text {max}},&0\le \mathfrak {Im}(\mu ) ,\\&0<\epsilon _{\text {min}}\le \mathfrak {Re}(\epsilon )\le \epsilon _{\text {max}},&0\le \mathfrak {Im}(\epsilon ),\\&0 \le \mathfrak {Re}(\kappa ^2),&0\le \mathfrak {Im}(\kappa ^2). \end{aligned}$$

We assume for simplicity that $\Omega _s$ has trivial cohomology, in other words that its first and second Betti numbers are zero [2, Sec. 4.4]. Qualitatively, this means that it doesn’t feature handles nor interior voids: it is homeomorphic to a ball.

### Remark 1

The hypothesis that the second Betti number is zero is only used to prove injectivity of the coupling problem for Hodge–Laplace operators. It can be dropped without any changes to the following development for couplings involving the Hodge–Helmholtz operator (*non-static* electromagnetic transmission problems). The hypothesis that the first Betti number is zero is used in Sect. 5 to guarantee the existence of a certain “scalar potential lifting” that greatly simplifies the Fredholm arguments.

Inside this possibly inhomogeneous isotropic physical body, the potential formulation of Maxwell’s equations in frequency domain driven by a source current ${\mathbf {J}}\in {\mathbf {L}}^{2}(\Omega _s)$ with angular frequency $\omega >0$ reads [12]

1a $$\begin{aligned} \mathbf {curl}\,\left( \mu ^{-1}({\mathbf {x}})\,\mathbf {curl}\,{\mathbf {U}}\right) +i\omega \epsilon ({\mathbf {x}}) \nabla V - \omega ^2 \epsilon ({\mathbf {x}})\,{\mathbf {U}}= & {} {\mathbf {J}}, \end{aligned}$$

1b $$\begin{aligned} \text {div}\,\left( \epsilon ({\mathbf {x}}){\mathbf {U}}\right) +i\omega V= & {} 0, \end{aligned}$$

where the Lorentz gauge (1b) relates the scalar potential *V* to the vector potential ${\mathbf {U}}$. Elimination of *V* using this relation leads to the Hodge–Helmholtz equation

2 $$\begin{aligned} \mathbf {curl}\,\left( \mu ^{-1}({\mathbf {x}})\,\mathbf {curl}\,{\mathbf {U}}\right) - \epsilon ({\mathbf {x}})\,\nabla \,\text {div}\, \left( \epsilon ({\mathbf {x}}){\mathbf {U}}\right) - \omega ^2\epsilon ({\mathbf {x}})\,{\mathbf {U}} = {\mathbf {J}}. \end{aligned}$$

Away from the source current, in the unbounded region $\Omega ':={\mathbb {R}}^3 \backslash {\overline{\Omega }}_s$ outside the scatterer $\Omega _s$, where we assume a homogeneous material with scalar constant permeability $\mu _0>0$ and dielectric permittivity $\epsilon _0>0$, Eq. (2) reduces to

$$\begin{aligned} -\Delta _{\eta }{\mathbf {U}}-\kappa ^2{\mathbf {U}}:=\mathbf {curl}\,\mathbf {curl}\,{\mathbf {U}} - \eta \,\nabla \,\text{ div }\,{\mathbf {U}} - \kappa ^2{\mathbf {U}} = 0, \end{aligned}$$

with constant coefficients $\eta = \mu _0\epsilon _0^2$ and $\kappa ^2 = \mu _0\epsilon _0\omega ^2$.

For given data ${\mathbf {g}}_R\in {\mathbf {H}}^{-1/2}(\text {div}_{\Gamma })$, $g_n\in H^{-1/2}(\Gamma )$, $\zeta _D\in H^{1/2}(\Gamma )$ and $\varvec{\zeta }_t\in {\mathbf {H}}^{-1/2}(\text {curl}_{\Gamma },\Gamma )$ on the boundary $\Gamma =\partial \Omega _s$, we are interested in the following transmission problem, cf. [21, Sec. 2.1.2], [12]: 
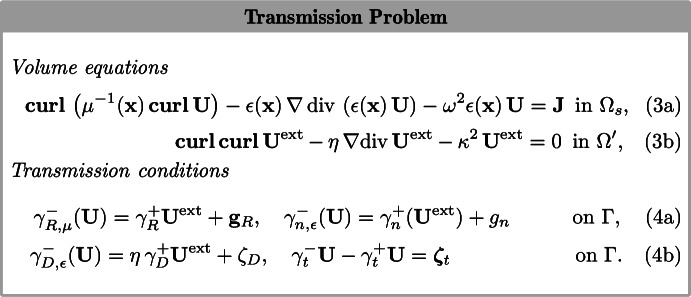


The traces $\gamma _{\bullet }^\mp$, $\bullet =R$, *D*, *n*, etc., on $\Gamma$ from inside (superscript −) and outside (superscript $+$) $\Omega _s$ are defined for a smooth vector-field ${\mathbf {U}}$ by

$$\begin{aligned}\ \gamma ^-_{R,\mu }({\mathbf {U}})&:= -\gamma ^-_{\tau }\left( \mu ^{-1}\left( {\mathbf {x}}\right) \,\mathbf {curl}({\mathbf {U}})\right) ,&\gamma ^+_{R}(\mathbf {U}^{\text{ ext }})&:= -\gamma ^+_{\tau }\left( \mathbf {curl}\left( \mathbf {U}^{\text{ ext }}\right) \right) ,\\ \gamma ^-_{D,\epsilon }({\mathbf {U}})&:=\gamma ^-\left( \text{ div }\left( \epsilon \left( {\mathbf {x}}\right) \,{\mathbf {U}}\right) \right) ,&\gamma ^+_{D}(\mathbf {U}^{\text{ ext }})&:=\gamma ^+\left( \text{ div }\left( \mathbf {U}^{\text{ ext }}\right) \right) ,\\ \gamma ^{-}_{n,\epsilon }({\mathbf {U}})&:= \gamma ^-_n(\epsilon \left( {\mathbf {x}}\right) \,{\mathbf {U}}),&\gamma ^\pm _{t}\left( {\mathbf {U}}\right)&:= {\mathbf {n}}\times \left( \gamma ^\pm _{\tau }\left( {\mathbf {U}}\right) \right) , \end{aligned}$$

involving the classical traces

$$\begin{aligned} \gamma \left( {\mathbf {U}}\right) := {\mathbf {U}}\big \vert _{\Gamma },&\gamma _n\left( {\mathbf {U}}\right) := \gamma \left( {\mathbf {U}}\right) \cdot {\mathbf {n}},&\gamma _{\tau }\left( {\mathbf {U}}\right) := \gamma \left( {\mathbf {U}}\right) \times {\mathbf {n}}, \end{aligned}$$

where ${\mathbf {n}}\in {\mathbf {L}}^{\infty }(\Gamma )$ is the essentially bounded unit normal vector field on $\Gamma$ directed toward the exterior of $\Omega _s$ [19, Thm. 3.1.6].

For positive frequencies $\omega >0$, we supplement (3a)–(4b) with the variants of the Silver-Müller’s radiation condition imposed at infinity provided in [21]. In the static case where $\kappa =\omega =0$, we seek a solution in an appropriate weighted Sobolev space that accounts for decay conditions [31, Sec. 2.5].

### Our Contributions

In the following, we couple the *mixed formulation* of the weak variational problem associated to (3a) with the first-kind boundary integral equation (BIE) arising from (3b) using these recently developed Calderón projectors for the Hodge–Helmholtz and Hodge–Laplace operators. The proof of the well-posedness of the coupled problem relies on T-coercivity (c.f. [14]) and is given in Sect. 5.2. It draws on and integrates several fundamental results of the theory of first-kind boundary integral operators on Lipschitz domains and of the mathematical analysis of Maxwell’s equations:

$$\triangleright $$
M. Costabel’s symmetric coupling approach linking volume variational equations with BIEs [17],
$$\triangleright $$
T-coercivity for electromagnetic variational problems via Hodge-type decompositions [15, 23],
$$\triangleright $$
mixed variational formulations of boundary value problems for Hodge–Laplace operators [3].

A crucial and surprising discovery is the perfect match of the interface terms naturally arising from the mixed variational formulation and from the first-kind BIE, see Sect. 3, and in particular (30), for details.

## Preliminaries

Let $\Omega \in \{\Omega _s,\Omega '\}$. As usual, $L^2(\Omega )$ and ${\mathbf {L}}^2(\Omega )$ denote the Hilbert spaces of square integrable scalar and vector-valued functions defined over $\Omega$. We denote their inner products using round brackets, e.g. $(\cdot ,\cdot )_{\Omega }$. Similarly, $H^1(\Omega )$ and ${\mathbf {H}}^1(\Omega )$ refer to the corresponding Sobolev spaces. We write $C^{\infty }_0(\Omega )$ for the space of smooth compactly supported functions in $\Omega$, but denote by ${\mathscr {D}}(\Omega )^3$ the analogous space of vector fields to simplify notation. The Banach spaces

$$\begin{aligned} {\mathbf {H}}(\text {div}, \Omega )&:=\{{\mathbf {U}}\in L^2(\Omega ) \,\vert \, \text {div}({\mathbf {U}})\in L^2(\Omega )\},\\ {\mathbf {H}}(\epsilon ;\text {div}, \Omega )&:=\{{\mathbf {U}}\in L^2(\Omega ) \,\vert \, \epsilon ({\mathbf {x}})\,{\mathbf {U}}\in {\mathbf {H}}(\text {div}, \Omega )\},\\ {\mathbf {H}}(\mathbf {curl}, \Omega )&:=\{{\mathbf {U}}\in L^2(\Omega ) \,\vert \, {\mathbf {curl}}\,({\mathbf {U}})\in L^2(\Omega )\},\\ {\mathbf {H}}\left( \nabla \text {div},\Omega \right)&:= \{{\mathbf {U}}\in {\mathbf {H}}\left( \text {div},\Omega \right) \,\vert \, \text {div}({\mathbf {U}})\in H^1(\Omega )\},\\ {\mathbf {H}}\left( \epsilon ;\nabla \text {div},\Omega _s\right)&:= \{{\mathbf {U}}\in {\mathbf {L}}^2(\Omega )\,\vert \,\epsilon ({\mathbf {x}})\,{\mathbf {U}}\in {\mathbf {H}}\left( \nabla \text {div},\Omega \right) \},\\ {\mathbf {H}}(\mathbf {curl}^2,\Omega )&:=\{{\mathbf {U}}\in {\mathbf {H}}(\mathbf {curl}, \Omega ) \,\vert \, \mathbf {curl}({\mathbf {U}})\in {\mathbf {H}}(\mathbf {curl}, \Omega )\},\\ {\mathbf {H}}(\mu ^{-1};\mathbf {curl}^2,\Omega )&:=\{{\mathbf {U}}\in {\mathbf {H}}(\mathbf {curl}, \Omega ) \,\vert \, \mu ^{-1}\,\mathbf {curl}({\mathbf {U}})\in {\mathbf {H}}(\mathbf {curl}, \Omega )\}, \end{aligned}$$

equipped with the natural graph norms will be important. The variational space for the primal variational formulation of the classical and generalized Hodge–Helmholtz/Laplace operator is given by

5 $$\begin{aligned} {\mathbf {X}}(\Delta ,\Omega )&:={\mathbf {H}}(\mathbf {curl}^2,\Omega )\cap {\mathbf {H}}\left( \nabla \text {div},\Omega \right) . \end{aligned}$$

A subscript is used to identify spaces of locally integrable functions or vector fields, e.g. $U\in L^2_{\text {loc}}(\Omega )$ if and only if $\phi U$ is square-integrable for all $\phi \in C^{\infty }_0({\mathbb {R}}^3)$. Dual spaces, e.g. $H^1_0(\Omega _s)'=H^{-1}(\Omega _s)$, and dual operators, e.g. $(\gamma ^-)'$, are written with primes. We use an asterisk to indicate spaces of functions with zero mean, e.g. $H^1_*(\Omega )$, and let $\mathbf {mean}:H^1(\Omega _s)\rightarrow {\mathbb {R}}$ be the continuous operator defined by

$$\begin{aligned} \mathbf {mean}(P):=\int _{\Omega _s}P({\mathbf {x}})\hbox {d}{\mathbf {x}}. \end{aligned}$$

Since its range is finite dimensional, $\mathbf {mean}$ is a compact operator [27, Thm. 2.18]. The operator $Q_*:H^1(\Omega _s)\rightarrow H^1_*(\Omega _s)$ defined by $Q_*=\mathrm {Id}-\mathbf {mean}$ is a projection onto mean zero functions.

### Trace Spaces

Development of trace-related theory for Lipschitz domains and detailed definitions for the surface differential operators $\nabla _{\Gamma }$, $\text {curl}_{\Gamma }$, $\mathbf {curl}_{\Gamma }$ and $\text {div}_{\Gamma }$ can be found in [7, 8, 10]. In this section, we define the product trace spaces required for a variational treatment of the Hodge–Laplace/Helmholtz operator. The traces are adapted to the system of equations at hand by accounting for the varying coefficients of (3a).

Based on the continuous and surjective extensions

$$\begin{aligned} \gamma&: H^1\left( \Omega \right) \rightarrow H^{1/2}\left( \Gamma \right) ,&\hbox {[26, Thm. 4.2.1]}\\ \gamma _n&: {\mathbf {H}}(\text {div}, \Omega )\rightarrow H^{-1/2}\left( \Gamma \right) ,&\hbox {[20, Thm. 2.5, Cor. 2.8]}\\ \gamma _{\tau }&:{\mathbf {H}}\left( \mathbf {curl},\Omega \right) \rightarrow {\mathbf {H}}^{-1/2}(\text {div}_\Gamma ,\Gamma ),&\hbox {[10, Thm. 4.1]}\\ \gamma _{t}&:{\mathbf {H}}\left( \mathbf {curl},\Omega \right) \rightarrow {\mathbf {H}}^{-1/2}(\text {curl}_\Gamma ,\Gamma ),&\hbox {[10, Thm. 4.1]} \end{aligned}$$

the traces previously introduced can also be extended by continuity to the relevant Sobolev spaces. We denote the duality pairing between $H^{1/2}(\Gamma )$ and $H^{-1/2}(\Gamma )$ by $\langle \cdot ,\cdot \rangle _{\Gamma }$, but use $\langle \cdot ,\cdot \rangle _{\tau }$ for the duality pairing between the trace spaces ${\mathbf {H}}^{-1/2}(\text {curl}_\Gamma ,\Gamma )$ and ${\mathbf {H}}^{-1/2}(\text {div}_\Gamma ,\Gamma )$ [10, Lem. 5.6].

The duality pairings enter Green’s formulas ($+$ for $\Omega =\Omega _s$)

6a $$\begin{aligned} \langle \gamma \left( P\right) \gamma _n\left( {\mathbf {W}}\right) \rangle _{\Gamma }= & {} \pm \int _{\Omega } \text {div}({\mathbf {W}})\, P + {\mathbf {W}}\cdot \nabla P \, \hbox {d}{\mathbf {x}}, \end{aligned}$$

6b $$\begin{aligned} \langle \gamma _{t}\left( {\mathbf {V}}\right) , \gamma _{\tau }\left( {\mathbf {U}}\right) \rangle _{\tau }= & {} \pm \int _{\Omega }{\mathbf {U}}\cdot \mathbf {curl\,}({\mathbf {V}}) -\mathbf {curl\,}({\mathbf {U}})\cdot {\mathbf {V}}\hbox {d}{\mathbf {x}}, \end{aligned}$$

6c $$\begin{aligned} \langle \gamma _t({\mathbf {V}}),\gamma _R({\mathbf {E}}) \rangle _{\tau }= & {} \pm \int _{\Omega }\mathbf {curl}\,\mathbf {curl}\,{\mathbf {E}}\cdot {\mathbf {V}} - \mathbf {curl}\,{\mathbf {E}}\cdot \mathbf {curl}\,{\mathbf {V}}\hbox {d}{\mathbf {x}}, \end{aligned}$$

which hold for all $P\in H^1(\Omega )$, ${\mathbf {W}}\in {\mathbf {H}}(\text {div},\Omega )$, ${\mathbf {U}},{\mathbf {V}}\in {\mathbf {H}}(\mathbf {curl},\Omega )$ and ${\mathbf {E}}\in {\mathbf {H}}(\mathbf {curl}^2,\Omega )$.

As explained in [15, Sec. 3], a theory of differential equations for the Hodge–Helmholtz/Laplace problem in three dimensions entails partitioning our collection of traces into two *dual* pairs. Accordingly, we now introduce the continuous and surjective mappings

$$\begin{aligned}&{\mathcal {T}}^{-}_{D,\epsilon }:{\mathbf {H}}_{\text {loc}}(\mathbf {curl},\Omega _s)\cap {\mathbf {H}}_{\text {loc}}(\epsilon ;\nabla \text {div},\Omega _s)\rightarrow {\mathcal {H}}_D(\Gamma ),\\&{\mathcal {T}}^{+}_{D}:{\mathbf {H}}_{\text {loc}}(\mathbf {curl},\Omega ')\cap {\mathbf {H}}_{\text {loc}}(\nabla \text {div},\Omega ')\rightarrow {\mathcal {H}}_D(\Gamma ),\\&{\mathcal {T}}^{-}_{N,\mu }:{\mathbf {H}}_{\text {loc}}(\mu ^{-1};\mathbf {curl}^2,\Omega _s)\cap {\mathbf {H}}_{\text {loc}}(\epsilon ;\text {div},\Omega _s) \rightarrow {\mathcal {H}}_N(\Gamma ),\\&{\mathcal {T}}^{+}_{N}:{\mathbf {H}}_{\text {loc}}(\mathbf {curl}^2,\Omega ')\cap {\mathbf {H}}_{\text {loc}}(\text {div},\Omega ') \rightarrow {\mathcal {H}}_N(\Gamma ), \end{aligned}$$

defined bywhere

$$\begin{aligned}&{\mathcal {H}}_D:={\mathbf {H}}^{-1/2}(\text {curl}_{\Gamma },\Gamma )\times H^{1/2}(\Gamma ),\\&{\mathcal {H}}_N:={\mathbf {H}}^{-1/2}(\text {div}_{\Gamma },\Gamma )\times H^{-1/2}(\Gamma ). \end{aligned}$$

They admit continuous right-inverses, i.e. lifting maps from the trace spaces into ${\mathbf {X}}(\Delta ,\Omega )$ [15, Lem. 3.2].

In literature the pair of traces involved in ${\mathcal {T}}_N$ is labelled as *magnetic*, while the pair in ${\mathcal {T}}_D$ is referred to as *electric*—simply because one recovers the magnetic field by taking the curl of the potential ${\mathbf {U}}$. However, our choice of subscripts is motivated by the analogy between this pair of product traces and the classical Dirichlet and Neumann boundary conditions for second-order elliptic BVPs.

The trace spaces ${\mathcal {H}}_D$ and ${\mathcal {H}}_N$ are put in duality using the sum of the inherited component-wise duality parings. That is, for $\vec {{\mathbf {p}}}=({\mathbf {p}},q)\in {\mathcal {H}}_N$ and $\vec {\varvec{\eta }}=(\varvec{\eta },\zeta )\in {\mathcal {H}}_D$, we define

$$\begin{aligned} \langle \vec {{\mathbf {p}}}, \vec {\varvec{\eta }}\rangle := \langle {\mathbf {p}},\varvec{\eta }\rangle _{\tau } + \langle q, \zeta \rangle _{\Gamma }. \end{aligned}$$

We indicate with curly brackets the average

$$\begin{aligned} \{\gamma _{\bullet }\}:= \frac{1}{2}\left( \gamma _{\bullet }^+ + \gamma _{\bullet }^-\right) \end{aligned}$$

of a trace and with square brackets its jump

$$\begin{aligned}{}[\gamma _{\bullet }]:=\gamma _{\bullet }^- - \gamma _{\bullet }^+ \end{aligned}$$

over the interface $\Gamma$, $\bullet =R$, *D*, *t*, $\tau$, or *n*. Corresponding notation is used for the product traces.

#### Warning

Notice the sign in the jump $[\gamma ]=\gamma ^- - \gamma ^+$, which is often taken to be the opposite in literature!

### Boundary Potentials

By exploiting the radiating fundamental solution

$$\begin{aligned} G_{\nu }({\mathbf {x}}):=\exp \left( i\nu |{{\mathbf {x}}}|\right) /4\pi |{{\mathbf {x}}}| \end{aligned}$$

for the scalar Helmholtz operator $-\Delta - \nu ^2\mathrm {Id}$, it is shown in [15, Sec. 4.2] that a distributional solution ${\mathbf {U}}\in {\mathbf {L}}^2({\mathbb {R}}^3)$ such that ${\mathbf {U}}\vert _{\Omega _s}\in {\mathbf {X}}(\Delta ,\Omega _s)$ and ${\mathbf {U}}\vert _{\Omega '}\in {\mathbf {X}}_{\text {loc}}(\Delta ,\Omega ')$ of the homogeneous (scaled) Hodge–Helmholtz/Laplace equation (3b) with constant coefficients $\eta >0$, $\kappa \ge 0$, stated in the whole of ${\mathbb {R}}^3$ with radiation conditions at infinity as considered in Sect. 1, affords a representation formula

7 $$\begin{aligned} {\mathbf {U}} = \mathcal {SL}_{\kappa }\cdot [{\mathcal {T}}_{N}({\mathbf {U}})] + \mathcal {DL}_{\kappa }\cdot [{\mathcal {T}}_{D}({\mathbf {U}})]\qquad \qquad \text {in }{\mathbb {R}}^3\backslash \Gamma . \end{aligned}$$

Letting ${\tilde{\kappa }}=\kappa /\sqrt{n}$, the Hodge–Helmholtz single layer potential is explicitly given by8where the Helmholtz scalar single-layer, vector single-layer and the regular potentials are written individually for ${\mathbf {p}}\in {\mathbf {H}}^{-1/2}(\text {div}_{\Gamma },\Gamma )$ and $q\in H^{-1/2}(\Gamma )$ as

9a $$\begin{aligned} \psi _{\nu }(q)({\mathbf {x}}):= & {} \int _{\Gamma }q({\mathbf {y}})G_{\nu }({\mathbf {x}}-{\mathbf {y}})\hbox {d}\sigma ({\mathbf {y}}), \qquad \qquad \quad {\mathbf {x}}\in {\mathbb {R}}^3\backslash \Gamma , \end{aligned}$$

9b $$\begin{aligned} \varvec{\Psi }_{\nu }({\mathbf {p}})({\mathbf {x}}):= & {} \int _{\gamma }{\mathbf {p}}({\mathbf {y}})G_{\nu }({\mathbf {x}}-{\mathbf {y}})\hbox {d}\sigma ({\mathbf {y}}), \qquad \qquad \quad {\mathbf {x}}\in {\mathbb {R}}^3\backslash \Gamma ,\end{aligned}$$

9c $$\begin{aligned} {\tilde{\psi }}_{\kappa }(q)({\mathbf {x}}):= & {} \int _{\Gamma }q({\mathbf {y}})\frac{G_{\kappa }-G_{{\tilde{\kappa }}}}{\kappa ^2}({\mathbf {x}}-{\mathbf {y}})\hbox {d}\sigma ({\mathbf {y}}), \,~\qquad {\mathbf {x}}\in {\mathbb {R}}^3\backslash \Gamma , \end{aligned}$$

respectively. The expression (8) is derived with (9a)–(9c) understood as duality pairings. However, if the essential supremum of ${\mathbf {p}}$, *q* and $\text {div}_{\Gamma }({\mathbf {p}})$ is bounded, then they can safely be computed as improper integrals [15, Rmk. 4.2]. These classical potentials satisfy

10a $$\begin{aligned} -\,\text {div}\,\nabla \psi _{{\tilde{\kappa }}}(q)= & {} {\tilde{\kappa }}^2\psi _{{\tilde{\kappa }}}(q), \end{aligned}$$

10b $$\begin{aligned} -\,\Delta \varvec{\Psi }_{\kappa }({\mathbf {p}})= & {} \kappa ^2\varvec{\Psi }_{\kappa }({\mathbf {p}}), \end{aligned}$$

10c $$\begin{aligned} -\text {div}\,\nabla {\tilde{\psi }}_{\kappa }(q)= & {} \psi _{\kappa }(q) + \frac{1}{\eta }\psi _{{\tilde{\kappa }}}(q), \end{aligned}$$

and the identity [28, Lem. 2.3]

11 $$\begin{aligned} \text {div}\,\varvec{\Psi }_{\nu }({\mathbf {p}}) = \psi _{\nu }\left( \text {div}_{\Gamma }{\mathbf {p}}\right) \qquad \qquad \forall \,{\mathbf {p}}\in {\mathbf {H}}^{-1/2}(\text {div}_\Gamma ,\Gamma ). \end{aligned}$$

The mapping properties of $\psi _{\nu }$, $\varvec{\Psi }_{\nu }$, $\nabla \psi _{{\tilde{\kappa }}}$ and $\nabla {\tilde{\psi }}_{\kappa }$ are detailed in [15, Sec. 5].

Ultimately, we will resort to a Fredholm alternative argument to prove well-posedness of the coupled system. It is therefore evident that the compactness properties of the boundary integral operators introduced in the next lemma will be extensively used both explicitly and implicitly—notably through exploiting the results found in [15, Sec. 6].

From [29, Lem. 3.9.8] and [11, Lem. 7], we know that for any $\nu \ge 0$, the following operators are compact:

12a $$\begin{aligned} \gamma ^{\pm }\left( \psi _{\nu }-\psi _{0}\right) :&\,H^{-1/2}(\Gamma )\rightarrow H^{1/2}(\Gamma ), \end{aligned}$$

12b $$\begin{aligned} \gamma ^{\pm }_n\left( \nabla \psi _{\nu }-\nabla \psi _{0}\right) :&\,H^{-1/2}(\Gamma )\rightarrow H^{-1/2}(\Gamma ),\end{aligned}$$

12c $$\begin{aligned} \gamma ^{\pm }_{t}\left( \varvec{\Psi }_{\nu }-\varvec{\Psi }_{0}\right) :&\,{\mathbf {H}}^{-1/2}(\text {div}_{\Gamma },\Gamma )\rightarrow {\mathbf {H}}^{-1/2}(\text {curl}_{\Gamma },\Gamma ),\end{aligned}$$

12d $$\begin{aligned} \gamma ^{\pm }_n\nabla {\tilde{\psi }}_{\nu }:&\,H^{-1/2}(\Gamma )\rightarrow H^{-1/2}(\Gamma ).\ \end{aligned}$$

Compactness of the second boundary integral operator listed immediately entails compactness of

$$\begin{aligned} \nu ^2\gamma ^{\pm }_n\nabla {\tilde{\psi }}_{\nu }=\gamma ^{\pm }_n\left( \nabla \psi _{\nu }-\nabla \psi _{{\tilde{\nu }}}\right) =\gamma ^{\pm }_n\left( \nabla \psi _{\nu }-\nabla \psi _{0}\right) - \left( \gamma ^{\pm }_n\left( \nabla \psi _{{\tilde{\nu }}}-\nabla \psi _{0}\right) \right) \end{aligned}$$

by linearity. While it seems that blow-up occurs in ${\tilde{\psi }}_{\nu }$ as $\nu \rightarrow 0$, $\nabla {\tilde{\psi }}_{\nu }$ happens to be an entire function of $\nu$ that vanishes at $\nu = 0$ [15, Sec. 4.1].

The Hodge–Helmholtz double layer potential is given for boundary data $\varvec{\eta }\in {\mathbf {H}}^{-1/2}(\text {curl}_{\Gamma },\Gamma )$ and $\xi \in H^{1/2}(\Gamma )$ by13We recognize in (13) the (electric) Maxwell double layer potential (c.f. [23, Sec. 4], [11, Eq. 28]) and the normal vector single-layer potential

$$\begin{aligned} \Upsilon _{\kappa }(\xi )&:=\int _{\Gamma }\xi ({\mathbf {y}}){\mathbf {G}}_{\kappa }({\mathbf {x}}-{\mathbf {y}}){\mathbf {n}}({\mathbf {y}})\hbox {d}\sigma ({\mathbf {y}}),&{\mathbf {x}}\in {\mathbb {R}}^3\backslash \Gamma , \end{aligned}$$

in which appears the matrix-valued fundamental solution

$$\begin{aligned} {\mathbf {G}}_{\kappa } := G_{\kappa }\mathrm {Id}+{\kappa ^{-2}}\nabla ^2\left( G_{\kappa }-G_{{\tilde{\kappa }}}\right) \end{aligned}$$

satisfying $-\Delta _{\eta }{\mathbf {G}}_{\kappa } - \kappa ^2{\mathbf {G}}_{\kappa }=\delta _0\,\mathrm {Id}$ exploited in [15] and detailed in [21, App. A]. This surface potential satisfies

14 $$\begin{aligned} -\Delta _{\eta }\Upsilon _{\kappa }(\xi )=\kappa ^2\Upsilon _{\kappa }(\xi ) \end{aligned}$$

and the identity [15, Sec. 5.4] $\mathbf {curl}\,\Upsilon _{\kappa }(\xi )=\mathbf {curl}\varvec{\Psi }_{\kappa }(\xi {\mathbf {n}})$.

The mapping properties of the potentials $\mathbf {curl}\varvec{\Psi }_{\kappa }(\cdot \times {\mathbf {n}})$ and $\Upsilon _{\kappa }$ are detailed in [15, Sec. 5].

### Integral Operators

In this section, we extend the analysis performed in [11, 23] for the classical electric wave equation to the boundary integral operators arising from Hodge–Helmholtz and Hodge–Laplace problems.

The well-known Caldéron identities are obtained from (7) upon taking the classical compounded traces on both sides and utilizing the jump relations

15a $$\begin{aligned}&[{\mathcal {T}}_D]\cdot \mathcal {DL}_{\kappa }(\vec {\varvec{\eta }}) = \vec {\varvec{\eta }}, \quad \quad [{\mathcal {T}}_N]\cdot \mathcal {DL}_{\kappa }(\vec {\varvec{\eta }}) = 0,\quad \quad \vec {\varvec{\eta }}\in {\mathcal {H}}_D, \end{aligned}$$

15b $$\begin{aligned}&[{\mathcal {T}}_D]\cdot \mathcal {SL}_{\kappa }(\vec {{\mathbf {p}}}) = 0, \quad \quad [{\mathcal {T}}_N]\cdot \mathcal {SL}_{\kappa }(\vec {{\mathbf {p}}}) = \vec {{\mathbf {p}}},\quad \quad \vec {{\mathbf {p}}}\in {\mathcal {H}}_N, \end{aligned}$$

given in [15, Thm. 5.1]. The operator forms of the interior and exterior Caldéron projectors defined on ${\mathcal {H}}_D\times {\mathcal {H}}_N$, which we denote ${\mathbb {P}}^-_{\kappa }$ and ${\mathbb {P}}^+_{\kappa }$ respectively, enter the Caldéron identites:

16a $$\begin{aligned}&\underbrace{\begin{pmatrix} \{{\mathcal {T}}_D\}\cdot \mathcal {DL}_k+\frac{1}{2}\mathrm {Id}&{} \{{\mathcal {T}}_D\}\cdot \mathcal {SL}_k\\ \{{\mathcal {T}}_N\}\cdot \mathcal {DL}_k &{} \{{\mathcal {T}}_N\}\cdot \mathcal {SL}_k+\frac{1}{2}\mathrm {Id}\end{pmatrix}}_{=:{\mathbb {P}}^-_{\kappa }} \begin{pmatrix} {\mathcal {T}}_D^-{\mathbf {U}}\\ {\mathcal {T}}_N^-{\mathbf {U}} \end{pmatrix} = \begin{pmatrix} {\mathcal {T}}_D^-{\mathbf {U}}\\ {\mathcal {T}}_N^-{\mathbf {U}} \end{pmatrix}, \end{aligned}$$

16b $$\begin{aligned}&\underbrace{\begin{pmatrix} -\{{\mathcal {T}}_D\}\cdot \mathcal {DL}_k+\frac{1}{2}\mathrm {Id}&{} -\{{\mathcal {T}}_D\}\cdot \mathcal {SL}_k\\ -\{{\mathcal {T}}_N\}\cdot \mathcal {DL}_k &{} -\{{\mathcal {T}}_N\}\cdot \mathcal {SL}_k+\frac{1}{2}\mathrm {Id}\end{pmatrix}}_{=:{\mathbb {P}}^+_{\kappa }} \begin{pmatrix} {\mathcal {T}}_D^+{\mathbf {U}}^{\text {ext}}\\ {\mathcal {T}}_N^+{\mathbf {U}}^{\text {ext}} \end{pmatrix} = \begin{pmatrix} {\mathcal {T}}_D^+{\mathbf {U}}^{\text {ext}}\\ {\mathcal {T}}_N^+{\mathbf {U}}^{\text {ext}} \end{pmatrix},\nonumber \\ \end{aligned}$$

Note that ${\mathbb {P}}^-_{\kappa } + {\mathbb {P}}^+_{\kappa }=\mathrm {Id}$ and that the range of ${\mathbb {P}}^+_{\kappa }$ coincides with the kernel of ${\mathbb {P}}^-_{\kappa }$ and vice–versa [11, Sec. 5]. As a consequence of the jump relations (15a)–(15b), the representation formula (7) and the existence of trace liftings, the pair of “magnetic” and “electric” traces $\left( \vec {\varvec{\eta }}\,\,\,\vec {{\mathbf {p}}}\right) ^{\top }\in {\mathcal {H}}_D\times {\mathcal {H}}_N$ is valid interior or exterior Cauchy data, if and only if it lies in the kernel of ${\mathbb {P}}^+_{\kappa }$ or ${\mathbb {P}}^-_{\kappa }$ respectively (c.f. [32, Lem. 6.18], [11, Thm. 8] and [15, Prop. 5.2]).

Inspecting Eqs. (16a)–(16b) reveals that the Caldéron projectors share a common structure. They can be written as

$$\begin{aligned} {\mathbb {P}}_{\kappa }^- = \frac{1}{2}\mathrm {Id}+ {\mathbb {A}}_\kappa&\text{ and }&{\mathbb {P}}_{\kappa }^+ = \frac{1}{2}\mathrm {Id}- {\mathbb {A}}_\kappa , \end{aligned}$$

and where the Caldéron operator ${\mathbb {A}}_\kappa :\mathcal {H_D\times {\mathcal {H}}_N\rightarrow {\mathcal {H}}_D\times {\mathcal {H}}_N}$ is given by

17 $$\begin{aligned} {\mathbb {A}}_\kappa :=\begin{pmatrix} {\mathbb {A}}_{\kappa }^{DD} &{} {\mathbb {A}}_{\kappa }^{ND}\\ {\mathbb {A}}_{\kappa }^{DN} &{} {\mathbb {A}}_{\kappa }^{NN} \end{pmatrix} := \begin{pmatrix} \{{\mathcal {T}}_D\}\cdot \mathcal {DL}_\kappa &{} \{{\mathcal {T}}_D\}\cdot \mathcal {SL}_\kappa \\ \{{\mathcal {T}}_N\}\cdot \mathcal {DL}_\kappa &{} \{{\mathcal {T}}_N\}\cdot \mathcal {SL}_\kappa \end{pmatrix}. \end{aligned}$$

An analog of the operator matrix ${\mathbb {A}}_k$ was found convenient in the study of the boundary integral equations of electromagnetic scattering problems [11, Sec. 6]. It is known from [15] that the off-diagonal blocks ${\mathbb {A}}_{\kappa }^{DN}$ and ${\mathbb {A}}_{\kappa }^{ND}$ of ${\mathbb {A}}_k$ independently satisfy generalized Gårding inequalities making them of Fredholm type with index 0. Injectivity holds when $\kappa ^2$ lies outside a discrete set of “forbidden resonant frequencies” accumulating at infinity [15, Sec. 3]. More explanations will be given in Section 3. In the static case $\kappa =0$, the dimensions of $\ker \left( \{{\mathcal {T}}_N\}\cdot \mathcal {SL}_0\right)$ and $\ker \left( \{{\mathcal {T}}_D\}\cdot \mathcal {DL}_0\right)$ agree with the zeroth and first Betti number of $\Gamma$, respectively [15, Sec. 7].

In the case of the classical electric wave equation, the boundary integral operators involved in the Caldéron projectors enjoy a hidden symmetry: there exists a compact linear operator ${\mathbf {C}}_k:{\mathbf {H}}^{-1/2}(\text {div}_\Gamma ,\Gamma )\rightarrow {\mathbf {H}}^{-1/2}(\text {div}_\Gamma ,\Gamma )$ such that

18 $$\begin{aligned} \langle \{\gamma _R\}\varvec{\Psi }_k({\mathbf {p}}), \varvec{\eta } \rangle _{\tau } = \langle {\mathbf {p}}, \{\gamma _t\}\varvec{\Psi }_{\kappa }\mathbf {curl}(\varvec{\eta }\times {\mathbf {n}}) \rangle _{\tau } +\langle {\mathbf {C}}_k{\mathbf {p}}, \varvec{\eta } \rangle _{\tau } \end{aligned}$$

for all ${\mathbf {p}}\in {\mathbf {H}}^{-1/2}(\text {div}_\Gamma ,\Gamma )$ and $\varvec{\eta }\in {\mathbf {H}}^{-1/2}(\mathbf {curl}_\Gamma ,\Gamma )$, cf. [23, Lem. 5.4] and [11, Lem. 6].

We will extend this result to the integral operators defined for the scaled Hodge–Helmholtz/Laplace equation to better characterize the structure of (17). The symmetry we are about to reveal in the diagonal blocks ${\mathbb {A}}_{\kappa }^{NN}$ and ${\mathbb {A}}_{\kappa }^{DD}$ of the Caldéron projectors will be crucial in the derivation of the main T-coercivity estimate of this work. It will be exploited for complete *cancellation*, *up to compact terms,* of the operators lying on the *off-diagonal* of the block operator matrix associated to the coupled variational system introduced in Sect. 3. The following lemmas are required.

#### Lemma 2.1

There is a compact linear operator $C_k:H^{-1/2}(\Gamma )\rightarrow H^{-1/2}(\Gamma )$ such that

$$\begin{aligned} \langle \{\gamma _n\}\nabla \psi _{{\tilde{\kappa }}}(q), \xi \rangle _{\Gamma } =-\langle q, \{\eta \,\gamma _D\}\Upsilon _{\kappa }(\xi ) \rangle _{\Gamma } + \langle C_kq, \xi \rangle _{\Gamma }, \end{aligned}$$

for all $$q\in H^{-1/2}(\Gamma )$$, $$\xi \in H^{1/2}(\Gamma )$$.

#### Proof

This proof utilizes a strategy found in [23, Lem. 5.4] and [9, Thm. 3.9]. Let $\rho > 0$ be such that $B_{\rho }$ is an open ball containing ${\overline{\Omega }}_s$. We will indicate with a hat (e.g. ${\widehat{\gamma }}$) the traces taken over the boundary $\partial B_{\rho }$ of that ball and use Green’s formula to compare the following terms.

On the one hand, using the scalar Helmholtz equation (10a) and recalling that ${\tilde{\kappa }}=\kappa /\sqrt{\eta }$, we have19and similarly,On the other hand, using (10a) together with the scaled Hodge–Helmholtz equation (14), we also have20Equations (19) and (20) together yieldSimilarly, the terms involving the exterior traces satisfyFrom the first row of the jump properties [15, Sec. 5]

21a $$\begin{aligned}&[\gamma _D]\nabla \psi _{{\tilde{\kappa }}}(q) = 0,\qquad \qquad [\gamma _n]\Upsilon _{\kappa }(\xi ) = 0, \end{aligned}$$

21b $$\begin{aligned}&[\gamma _D]\Upsilon _{\kappa }(\xi ) = \xi /\eta , \qquad \qquad [\gamma _n]\nabla \psi _{{\tilde{\kappa }}}(q)=q , \end{aligned}$$

we obtain, by gathering the above results, integrating by parts again and using the fact that $\mathbf {curl}\circ \nabla \equiv 0$,22Fortunately, when restricted to domains away from $\Gamma$, the potentials are $C^{\infty }$-smoothing. Hence, their evaluation on $\partial B_{\rho }$, the highlighted terms in (22), induce compact operators. This shows that for some compact operator $C_k:H^{-1/2}(\Gamma )\rightarrow H^{-1/2}(\Gamma )$,

23 $$\begin{aligned} \langle \gamma _n^-\nabla \psi _{{\tilde{\kappa }}}(q), \eta \,\gamma _D^-\Upsilon _{\kappa }(\xi )\rangle _{\Gamma } = \langle \gamma _n^+\nabla \psi _{{\tilde{\kappa }}}(q), \eta \,\gamma _D^+\Upsilon _{\kappa }(\xi )\rangle _{\Gamma } + \langle C_k q, \xi \rangle _{\Gamma }. \end{aligned}$$

The jump identities (21b) for the potentials yield formulas of the form $\{\gamma _{\bullet }\}K = \gamma _{\bullet }^{\pm }K\pm (1/2)\mathrm {Id}$, where $\bullet =n,\,D$ and $K=\nabla \psi _{{\tilde{\kappa }}},\,\Upsilon _{\kappa }$ accordingly. Substituting each one-sided trace involved in the two leftmost duality pairings of (23) for the integral operators using these equations completes the proof.$\square$

#### Lemma 2.2

For all ${\mathbf {p}}\in {\mathbf {H}}^{-1/2}(\mathrm{div} _\Gamma ,\Gamma )$ and $\xi \in H^{1/2}(\Gamma )$, we have

$$\begin{aligned} \langle {\mathbf {p}}, \gamma ^{\pm }_t\Upsilon _{\kappa }(\xi ) \rangle _{\tau } = \langle \gamma ^{\pm }_n\varvec{\Psi }_{\kappa }({\mathbf {p}}),\xi \rangle _{\Gamma } + \langle \gamma ^{\pm }_n\nabla {\tilde{\psi }}_{\kappa }(\text {div }_{\Gamma }({\mathbf {p}})), \xi \rangle _{\Gamma }. \end{aligned}$$

#### Proof

In the following calculations, the boundary integrals are to be understood as duality pairings. Since ${\mathbf {p}}\in {\mathbf {L}}^2_t(\Gamma )$ is a tangent vector field lying in the image of $\gamma _t$, the tangential trace operator can safely be dropped in expanding these integrals using the definitions of Sect. 2.2. On the one hand, this leads towhere ${\tilde{G}}_{\kappa }:=\left( G_{\kappa }-G_{{\tilde{\kappa }}}\right) /\kappa ^2$.

On the other hand, the same observation implies that $\langle {\mathbf {p}},\nabla _\Gamma \gamma {\mathbf {V}})\rangle _{\tau }=\langle {\mathbf {p}},\gamma \nabla {\mathbf {V}})\rangle _{\tau }$ for any ${\mathbf {V}}\in {\mathbf {H}}_{\text {loc}}^1({\mathbb {R}}^3)$, and thus thatwhere we have remembered that the tangential divergence defined in Sect. 2.1 was adjoint to the negative surface gradient. Recognizing the Helmholtz vector single-layer potential in the first expression on the right hand side concludes the proof. $\square$

#### Proposition 2.3

There exists a compact operator ${\mathcal {C}}_k:{\mathcal {H}}_N\rightarrow {\mathcal {H}}_N$ such that

$$\begin{aligned} \langle {\mathbb {A}}^{NN}_\kappa (\vec {{\mathbf {p}}}), \vec {\varvec{\eta }} \rangle = -\langle \vec {{\mathbf {p}}}, {\mathbb {A}}^{DD}_{\kappa }(\vec {\varvec{\eta }}) \rangle + \langle {\mathcal {C}}_k\vec {{\mathbf {p}}}, \vec {\varvec{\eta }} \rangle \end{aligned}$$

for all $\vec {\varvec{\eta }}:=\left( \varvec{\eta },\,\, \xi \right) ^{\top }\in {\mathcal {H}}_D$ and $\vec {{\mathbf {p}}}:=({\mathbf {p}},\,\, q)^{\top }\in {\mathcal {H}}_N$.

#### Proof

Recall that ${\mathbb {A}}^{NN}_{\kappa } = \{{\mathcal {T}}_N\}\cdot {\mathcal {S}}{\mathcal {L}}_{\kappa }$ and ${\mathbb {A}}^{DD}_{\kappa } = \{{\mathcal {T}}_D\}\cdot {\mathcal {D}}{\mathcal {L}}_{\kappa }$. Since $\mathbf {curl}\circ \nabla =0$, $\langle \{\gamma _R\}\nabla \psi _{{\tilde{k}}}(q),\varvec{\eta }\rangle _{\tau } = 0$ and $\langle \{\gamma _R\}\nabla {\tilde{\psi }}_{k}(\text {div}_{\Gamma }({\mathbf {p}})),\varvec{\eta }\rangle _{\tau } = 0$; therefore,

24 $$\begin{aligned} \begin{aligned} \langle \{{\mathcal {T}}_{N}\}\cdot \mathcal {SL}_k(\vec {{\mathbf {p}}}), \vec {\varvec{\eta }} \rangle&= \langle -\{\gamma _R\}\varvec{\Psi }_{\kappa }({\mathbf {p}}),\varvec{\eta }\rangle _{\tau } + \langle \{\gamma _n\}\nabla \psi _{{\tilde{\kappa }}}(q), \xi \rangle _{\Gamma }\\&\quad - \langle \{\gamma _n\}\varvec{\Psi }_{\kappa }({\mathbf {p}}),\xi \rangle _{\Gamma } -\langle \{\gamma _n\}\nabla {\tilde{\psi }}_{\kappa }(\text {div}_{\Gamma }({\mathbf {p}})), \xi \rangle _{\Gamma }.\qquad \end{aligned} \end{aligned}$$

Since $\text {div}\circ \mathbf {curl}=0$, we also have $\{\gamma _D\}\,\mathbf {curl}\varvec{\Psi _{\kappa }} =0$. Hence, we need to compare (24) with

$$\begin{aligned} \langle \vec {{\mathbf {p}}}, \{{\mathcal {T}}_{D}\}\cdot \mathcal {DL}_k(\vec {\varvec{\eta }}) \rangle&=\langle {\mathbf {p}}, \{\gamma _t\}\mathbf {curl}\varvec{\Psi }_k(\varvec{\eta }\times {\mathbf {n}}) \rangle _{\tau } + \langle q, \{\eta \,\gamma _D\}\Upsilon _{\kappa }(\xi ) \rangle _{\Gamma }\\&\quad + \langle {\mathbf {p}}, \{\gamma _t\}\Upsilon _{\kappa }(\xi ) \rangle _{\tau }. \end{aligned}$$

The desired result follows by combining the known symmetry result from (18) with Lemma 2.1 and Lemma 2.2. $\square$

As consequence of Proposition 2.3, we have

$$\begin{aligned} \left( {\mathbb {P}}^+_{\kappa }\right) _{11}' \hat{=}\left( {\mathbb {P}}^-_{\kappa }\right) _{22}, \end{aligned}$$

where $\hat{=}$ is used to indicate equality up to compact terms.

## Coupled Problem

In this section, we derive a variational formulation for the system (3a)–(4b) which couples a mixed variational formulation defined in the interior domain to a boundary integral equation of the first kind that arises in the exterior domain.

As proposed in [3], we introduce a new variable $P= -\text {div}\left( \epsilon ({\mathbf {x}}){\mathbf {U}}\right)$ into Eq. (3a) to dispense with trial spaces contained in ${\mathbf {H}}(\mathbf {curl},\Omega _s)\cap {\mathbf {H}}(\text {div},\Omega _s)$. Applying Green’s formula (6c) in $\Omega _s$, we obtain

25 $$\begin{aligned} \begin{aligned}&\int _{\Omega _s}\mu ^{-1}\,\mathbf {curl}\,{\mathbf {U}}\cdot \mathbf {curl}\,{\mathbf {V}}\hbox {d}{\mathbf {x}} + \int _{\Omega _s}\epsilon \,\nabla P\cdot {\mathbf {V}}\hbox {d}{\mathbf {x}} \qquad {} \\&\quad -\omega ^2\int _{\Omega _s}\epsilon \,{\mathbf {U}}\cdot {\mathbf {V}}\hbox {d}{\mathbf {x}} +\langle \gamma _{R,\mu }^{-}{\mathbf {U}},\gamma ^-_t{\mathbf {V}}\rangle _{\tau } = \left( {\mathbf {J}},{\mathbf {V}}\right) _{\Omega _s},\\&\int _{\Omega _s}P\,Q\hbox {d}{\mathbf {x}} - \int _{\Omega _s}\epsilon \,{\mathbf {U}}\cdot \nabla Q \hbox {d}{\mathbf {x}} +\langle \gamma ^{-}_{n,\epsilon }{\mathbf {U}},\gamma ^{-} Q\rangle _{\Gamma }= 0 \end{aligned} \end{aligned}$$

for all ${\mathbf {V}}\in {\mathbf {H}}\left( \mathbf {curl}, \Omega _s\right)$, $Q\in H^1(\Omega _s)$. The volume integrals in these equations enter the interior bi-linear form26related to the one supplied for the Hodge–Laplace operator in [4, Sec. 3.2]. We aim to couple (26) with the BIEs replacing the PDEs in $\Omega '$. We use the transmission conditions (4a)–(4b) to couple (25) to the variational equationwhich involves a functional

$$\begin{aligned} {\mathscr {G}}\left( ({\mathbf {V}}\, Q)^{\top }\right) := ({\mathbf {J}},{\mathbf {V}})_{\Omega _s} - \langle ({\mathbf {g}}_R\,g_n)^{\top },(\gamma ^{-}_t{\mathbf {V}}\,\gamma ^{-} Q)^{\top }\rangle \end{aligned}$$

bounded over the test space. The exterior Calderón projector can be used to express the so-called Dirichlet-to-Neumann operator in two different ways.

1. Introducing the jump conditions into the *first exterior Calderón identity* given on the first line of (16b) along with a new unknown $\vec {{\mathbf {p}}}={\mathcal {T}}^+_{N} ({\mathbf {U}}^{\text {ext}})$ yields a variational system27for all $({\mathbf {V}}\,Q)^{\top }\in {\mathbf {H}}\left( \mathbf {curl}, \Omega _s\right) \times H^1(\Omega _s)$ and $\vec {{\mathbf {a}}}\in {\mathcal {H}}_N$, resembling the original Johnson-Nedélec coupling [6]. The new functional appearing on the right hand side of (27) is defined as

28 $$\begin{aligned} {\mathscr {R}}\left( \vec {{\mathbf {a}}}\right) : = \langle \left( \{{\mathcal {T}}_{D}\}\cdot \mathcal {DL}_{\kappa }+\frac{1}{2}\mathrm {Id}\right) (\varvec{\zeta }_t,\,\zeta _D)^{\top }, \vec {{\mathbf {a}}}\rangle . \end{aligned}$$

2. Following the exposition of Costabel in [17], we also retain the *second exterior Calderón identity* —in which we again introduce the jump conditions to eliminate the dependence on the exterior solution— and insert the resulting Eq. (27) to obtain the symmetrically coupled problem. Again, the right hand side of our system of equations has to be modified to include a new bounded linear functional

29 $$\begin{aligned} {\mathscr {F}}(\vec {{\mathbf {V}}}):= {\mathscr {G}}(V) + \langle -\{{\mathcal {T}}_{N}\}\cdot \mathcal {DL}_{\kappa }(\varvec{\zeta }_t,\zeta _D)^{\top },(\gamma ^{-}_t{\mathbf {V}},\,\gamma ^{-} Q)^{\top }\rangle . \end{aligned}$$

We arrive at the following variational problem. 
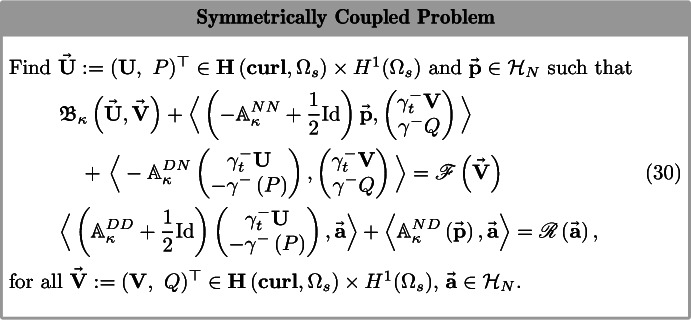


### Remark 2

Part of the justification for using mixed formulations for problems involving the Hodge–Helmholtz/Laplace operator is the need to avoid trial spaces contained in ${\mathbf {H}}(\mathbf {curl},\Omega _s)\cap {\mathbf {H}}(\text {div},\Omega _s)$, because the latter doesn’t allow for viable discretizations using finite elements [4]. While from (27) the issue seems to reappear after using the Caldéron identities, the benefits of the introduced new unknown $P\in H^1(\Omega _s)$ in the mixed formulation conveniently carries over to the coupled system (30) upon substituting $-\gamma ^-\left( P\right)$ in place of $\gamma _{D,\epsilon }({\mathbf {U}})$ in ${\mathcal {T}}^-_{D,\epsilon }({\mathbf {U}})$.

In the following proposition, we call *forbidden resonant frequencies* the interior “Dirichlet” (or electric) eigenvalues of the scaled Hodge–Laplace operator with constant coefficient $\eta = \mu _0\epsilon _0^2$. That is, $\kappa ^2$ is a forbidden frequency if there exists a *non-trivial* solution ${\mathbf {U}}\ne 0$ in ${\mathbf {X}}(\Delta ,\Omega )$ to

$$\begin{aligned} -\Delta _{\eta }{\mathbf {U}}-\kappa ^2{\mathbf {U}}&= 0,&\text{ in } \Omega _s,\\ {\mathcal {T}}^{-}_D{\mathbf {U}}&=0,&\text{ on } \Gamma . \end{aligned}$$

We refer the reader to [15], where the spectrum of the scaled Hodge–Laplace operator is completely characterized. See for e.g. [13, 16, 18, 29, 30] for an overview of the issue of spurious resonances in electromagnetic and acoustic scattering models based on integral equations.

### Proposition 3.1

Suppose that $\kappa ^2\in {\mathbb {C}}$ avoids forbidden resonant frequencies. By retaining an interior solution $U\in {\mathbf {H}}\left( \mathbf {curl}, \Omega _s\right)$ and producing ${\mathbf {U}}^{\text {ext}}\in {\mathbf {X}}_{\text {loc }}(\Delta ,\Omega ')$ using the representation formula (7) for the obtained Cauchy data $(\vec {{\mathbf {p}}},{\mathcal {T}}_{D,\epsilon }^-U - (\varvec{\zeta }_t,\,\,\zeta _D)^{\top })$ with $\gamma ^-_{D,\epsilon }({\mathbf {U}}) = -\gamma ^-\left( P\right)$, a solution to (30) solves the transmission system (3a)–(4b) in the sense of distribution.

### Proof

The proof follows the approach in [23, Lem. 6.1]. Since ${\mathscr {D}}(\Omega _s)^3\times C^{\infty }_0(\Omega _s)$ is a subset of the volume test space, any solution to the problem (30) solves (3a) in $\Omega _s$ in the sense of distribution. It follows that (25) holds for all admissible $\vec {{\mathbf {V}}}$, which reduces (30) to the variational systemwhere $\vec {{\mathbf {q}}} := {\mathcal {T}}_{N,\mu }^-({\mathbf {U}}) - ({\mathbf {g}}_R,\,\,g_n)^{\top }$ and $\vec {\varvec{\xi }}:={\mathcal {T}}^-_{D,\epsilon }({\mathbf {U}})-(\varvec{\zeta }_t,\,\,\zeta _D)^{\top }$.

We recognize in the equivalent operator equation

31 $$\begin{aligned} \underbrace{\begin{pmatrix} {\mathbb {A}}^{NN}_{\kappa }+\frac{1}{2}\mathrm {Id}&{} {\mathbb {A}}^{DN}_{\kappa }\\ {\mathbb {A}}^{ND}_{\kappa } &{} {\mathbb {A}}^{DD}_{\kappa }+\frac{1}{2}\mathrm {Id}\end{pmatrix}}_{{\mathbb {P}}^-_{\kappa }} \begin{pmatrix} \vec {{\mathbf {p}}}\\ \vec {\varvec{\xi }} \end{pmatrix} = \begin{pmatrix} \vec {{\mathbf {p}}}-\vec {{\mathbf {q}}}\\ 0 \end{pmatrix} \end{aligned}$$

the interior Caldéron projector (16a) whose image is the space of valid Cauchy data for the homogeneous (scaled) Hodge–Laplace/Helmholtz interior equation with constant coefficient $\eta$. In particular, $\vec {{\mathbf {p}}}-\vec {{\mathbf {q}}}={\mathcal {T}}^-_N\left( \tilde{{\mathbf {U}}}\right)$ for some vector-field $\tilde{{\mathbf {U}}}\in {\mathbf {X}}\left( \Delta ,\Omega _s\right)$ satisfying

32 $$\begin{aligned} \begin{aligned} -\Delta _{\eta } \tilde{{\mathbf {U}}} - \kappa ^2 \tilde{{\mathbf {U}}}&= 0, \qquad \qquad \qquad \text{ in } \Omega _s\\ {\mathcal {T}}^-_D\left( \tilde{{\mathbf {U}}}\right)&= 0, \qquad \qquad \qquad \text{ on } \Gamma . \end{aligned} \end{aligned}$$

If $\kappa ^2\ne 0$, we rely on the hypothesis that $\kappa ^2$ doesn’t belong to the set of forbidden resonant frequencies to guarantee injectivity of the above boundary value problem [15, Sec. 3] [21, Sec. 3]. Otherwise, the second Betti number of $\Omega _s$ being zero implies that zero is not a Dirichlet eigenvalue [2, Sec. 4.5.3]. We conclude that $\tilde{{\mathbf {U}}}=0$ is the unique trivial solution to (32). Therefore, for the right hand side of (31) to exhibit valid Neumann data, it must be that $\vec {{\mathbf {p}}}=\vec {{\mathbf {q}}}$.

Now, the null space of the interior Caldéron projector ${\mathbb {P}}^-_{\kappa }$ coincides with valid Cauchy data for the exterior boundary value problem (3b) complemented with the radiation conditions at infinity introduced in Sect. 1. In particular $(\vec {{\mathbf {p}}},\,\,\vec {\varvec{\xi }})^{\top }$ is valid Cauchy data for that exterior Hodge–Helmholtz or Hodge–Laplace problem and ${\mathbf {U}}^{\text {ext}}=\mathcal {SL}_{\kappa }\left( \vec {{\mathbf {p}}}\right) + \mathcal {DL}_{\kappa }\left( \vec {\varvec{\xi }}\right)$ solves (3b) and (4b) by construction. The fact that $\vec {{\mathbf {p}}}={\mathcal {T}}^+_{N}\left( {\mathbf {U}}^{\text {ext}}\right)$ solves (4a) is confirmed by the earlier observation that $\vec {{\mathbf {p}}}=\vec {{\mathbf {q}}}$. $\square$

### Corollary 3.2

Suppose that $\kappa ^2\in {\mathbb {C}}$ avoids forbidden resonant frequencies. A solution pair $\left( \vec {{\mathbf {U}}},\,\vec {{\mathbf {p}}}\right)$ to the coupled problem (30) is unique.

### Remark 3

We show in [30], where the kernel of the coupled problem is completely characterized, that when $\kappa ^2$ happens to be a resonant frequency, the interior solution ${\mathbf {U}}$ remains unique. This is no longer true for $\vec {{\mathbf {p}}}$ however, which is in general only unique up to Neumann traces of interior Dirichlet eigenfunctions of $-\Delta _{\eta }$ associated to the eigenvalue $\kappa ^2$. Fortunately, this kernel vanishes under the exterior representation formula obtained from (7).

## Space Decompositions

Using the classical Hodge decomposition, a general inf-sup condition for Hodge–Laplace problems posed on closed Hilbert complexes was derived in [4]. However, as orthogonality won’t be important, we rather opt for the enhanced regularity of the regular decomposition proposed in [11, 15]. There, a continuous projection ${\mathsf {Z}}:{\mathbf {H}}\left( \mathbf {curl},\Omega _s\right) \rightarrow {\mathbf {H}}^1(\Omega _s)$ is defined such that $\ker \left( {\mathsf {Z}}\right) =\ker \left( \mathbf {curl}\right) \cap {\mathbf {H}}\left( \mathbf {curl},\Omega _s\right)$ and $\mathbf {curl}\left( {\mathsf {Z}}({\mathbf {U}})\right) =\mathbf {curl}\left( {\mathbf {U}}\right)$. From Rellich’s theorem, this operator is compact as a mapping ${\mathsf {Z}}:{\mathbf {H}}\left( \mathbf {curl},\Omega _s\right) \rightarrow {\mathbf {L}}^2(\Omega _s)$. Therefore, a stable direct regular decomposition

33 $$\begin{aligned} {\mathbf {H}}\left( \mathbf {curl},\Omega _s\right) = {\mathbf {X}}(\mathbf {curl},\Omega _s) \oplus {\mathbf {N}}\left( \mathbf {curl},\Omega _s\right) . \end{aligned}$$

is provided by defining the subspaces ${\mathbf {X}}(\mathbf {curl},\Omega _s):={\mathsf {Z}}\left( {\mathbf {H}}\left( \mathbf {curl},\Omega _s\right) \right)$ and ${\mathbf {N}}\left( \mathbf {curl},\Omega _s\right) :=\ker \left( \mathbf {curl}\right) \cap {\mathbf {H}}\left( \mathbf {curl},\Omega _s\right)$.

A decomposition with similar properties can be designed for the space ${\mathbf {H}}^{-1/2}\left( \text {div}_{\Gamma },\Gamma \right)$ with a projection operator ${\mathsf {Z}}^{\Gamma }:{\mathbf {H}}^{-1/2}\left( \text {div}_{\Gamma },\Gamma \right) \rightarrow {\mathbf {H}}^{1/2}_R(\Gamma )$ satisfying $\ker ({\mathsf {Z}}^{\Gamma })=\ker \left( \text {div}_{\Gamma }\right) \cap {\mathbf {H}}^{-1/2}\left( \text {div}_{\Gamma },\Gamma \right)$ and $\text {div}_{\Gamma }\left( {\mathsf {Z}}^{\Gamma }({\mathbf {p}})\right) =\text {div}_{\Gamma }\left( {\mathbf {p}}\right)$.

As before, the extra regularity of the range, in this case provided by [23, Lem. 3.2], leads to compactness of the mapping ${\mathsf {Z}}^{\Gamma }:{\mathbf {H}}^{-1/2}\left( {\text {div}}_{\Gamma },\Gamma \right) \rightarrow {\mathbf {H}}^{-1/2}_R(\Gamma )$.

The subspaces ${\mathbf {X}}\left( \text {div}_{\Gamma },\Gamma \right) :={\mathsf {Z}}^{\Gamma }\left( {\mathbf {H}}^{-1/2}\left( \text {div}_{\Gamma },\Gamma \right) \right)$ and ${\mathbf {N}}\left( \text {div}_{\Gamma },\Gamma \right) :=\ker \left( \text {div}_{\Gamma }\right) \cap {\mathbf {H}}^{-1/2}\left( \text {div}_{\Gamma },\Gamma \right)$ provide a stable direct regular decomposition

34 $$\begin{aligned} {\mathbf {H}}^{-1/2}\left( \text {div}_{\Gamma },\Gamma \right) = {\mathbf {X}}\left( \text {div}_{\Gamma },\Gamma \right) \oplus {\mathbf {N}}\left( \text {div}_{\Gamma },\Gamma \right) . \end{aligned}$$

In the following, we may simplify notation by using ${\mathbf {U}}^{\perp }:={\mathsf {Z}}{\mathbf {U}}$, ${\mathbf {p}}^{\perp }:={\mathsf {Z}}^{\Gamma }{\mathbf {p}}$, ${\mathbf {U}}^0:=\left( \mathrm {Id}-{\mathsf {Z}}\right) {\mathbf {U}}$ and ${\mathbf {p}}^0:=\left( \mathrm {Id}-{\mathsf {Z}}^{\Gamma }\right) {\mathbf {p}}$.

A very useful property of this pair of decompositions is stated is shown in [23, Lem. 8.1] and [23, Lem. 8.2]: The operators

35a $$\begin{aligned} \left( \gamma _t^{-}\right) '\circ \left( \{\gamma _R\}\varvec{\Psi }_{\kappa }+\frac{1}{2}\mathrm {Id}\right) :{\mathbf {N}}\left( {\text {div}}_{\Gamma },\Gamma \right) \rightarrow {\mathbf {N}}\left( \mathbf {curl},\Omega _s\right) ', \end{aligned}$$

and

35b $$\begin{aligned} \left( \gamma _t^{-}\right) '\circ \left( \{\gamma _R\}\varvec{\Psi }_{\kappa }+\frac{1}{2}\mathrm {Id}\right) :{\mathbf {X}}\left( {\text {div}}_{\Gamma },\Gamma \right) \rightarrow {\mathbf {X}}\left( \mathbf {curl},\Omega _s\right) ' \end{aligned}$$

are compact.

Another benefit of this pair of regular decompositions will become explicit in the poof of Lemma 5.9 found in the next section.

It follows from [15, Lem. 6.4] that $\text {div}_{\Gamma }:{\mathbf {X}}\left( \text {div}_{\Gamma },\Gamma \right) \rightarrow H^{-1/2}_*(\Gamma )$ is a continuous bijection. The bounded inverse theorem guarantees the existence of a continuous inverse $\left( \text {div}_{\Gamma }\right) ^{\dag }:H^{-1/2}_*(\Gamma )\rightarrow {\mathbf {X}}\left( \text {div}_{\Gamma },\Gamma \right)$ such that

$$\begin{aligned} \left( \text {div}_{\Gamma }\right) ^{\dag }\circ \text {div}_{\Gamma }&=\mathrm {Id}\Big \vert _{{\mathbf {X}}\left( \text {div}_{\Gamma },\Gamma \right) },&\text {div}_{\Gamma }\circ \left( \text {div}_{\Gamma }\right) ^{\dag }=\mathrm {Id}\Big \vert _{H^{-1/2}_*(\Gamma )}. \end{aligned}$$

## Well-Posedness of the Coupled Variational Problem

We use the direct decompositions introduced in Sect. 4 to prove that the bilinear form associated to the coupled system (3.1) of Sect. 3 satisfies a generalized Gårding inequality.

The coupled variational problem (30) translates into the operator equationLetting ${\mathsf {B}}_{\kappa }:{\mathbf {H}}\left( \mathbf {curl},\Omega _s\right) \times H^1\left( \Omega _s\right) \rightarrow \left( {\mathbf {H}}\left( \mathbf {curl},\Omega _s\right) \times H^1\left( \Omega _s\right) \right) '$ be the operator

$$\begin{aligned}\langle {\mathsf {B}}_{\kappa }\left( \vec {{\mathbf {U}}}\right) \vec {{\mathbf {V}}}\rangle :={\mathfrak {B}}_{\kappa }\left( \vec {{\mathbf {U}}},\vec {{\mathbf {V}}}\right) \end{aligned}$$

associated with the Hodge–Helmholtz/Laplace volume contribution to the system, the operator

$$\begin{aligned} {\mathbb {G}}_{\kappa }:\left( {\mathbf {H}}\left( \mathbf {curl},\Omega _s\right) \times H^1(\Omega )\right) \times {\mathcal {H}}_N\rightarrow \left( {\mathbf {H}}\left( \mathbf {curl},\Omega _s\right) \times H^1(\Omega )\right) '\times \left( {\mathcal {H}}_N\right) ' \end{aligned}$$

can be represented by the block operator matrixshown here in “variational arrangement”.

The symmetry revealed in Sect. 2.3 makes explicit much of the structure of the above operator. We have introduced colors to better highlight the contribution of each individual block in the following sections.

Our goal is to design an isomorphism ${\mathbb {X}}$ of the test space and resort to compact perturbations of ${\mathbb {G}}_{\kappa }\circ {\mathbb {X}}^{-1}$ to achieve an operator block structure with diagonal blocks that are elliptic over the splittings of Sect. 4 and off-diagonal blocks that fit a skew-symmetric pattern. Stability of the coupled system can then be obtained from the next theorem. An overline indicates component-wise complex conjugation.

### Theorem 5.1

([11, Thm. 4]). If a bilinear form $a:V\times V\rightarrow {\mathbb {C}}$ on a reflexive Banach space *V* is T-coercive:

36 $$\begin{aligned} |{a\left( u,{\mathbb {X}}{\overline{u}}\right) + c\left( u,{\overline{u}}\right) }|\ge C\Vert u\Vert ^2_V\quad \forall u\in V, \end{aligned}$$

with $C>0$, $c:V\times V\rightarrow {\mathbb {C}}$ compact and ${\mathbb {X}}:V\rightarrow V$ an isomorphism of *V*, then the operator $A:V\rightarrow V'$ defined by $A:u\mapsto a(u,\cdot )$ is Fredholm with index 0.

The authors of [9] refer to (36) as “Generalized Gårding inequality”, because

$$\begin{aligned} |{a\left( u,{\mathbb {X}}{\overline{u}}\right) }|\ge C\Vert u\Vert ^2_V -|{c\left( u,{\overline{u}}\right) }|\qquad \forall \,u\in V, \end{aligned}$$

generalizes the classical Gårding inequality for a bilinear form *b* associated with uniformly elliptic operator of even order $2\ell$: $\exists \, C_2\ge 0, C_1>0$ such that

$$\begin{aligned} b(u,u) \ge C_1\Vert u\Vert ^2_{H^\ell (\Omega )}- C_2\Vert u\Vert _{L^2\left( \Omega \right) }\qquad \forall \,u\in H^\ell _0(\Omega ). \end{aligned}$$

Assuming that (36) holds with ${\mathbb {X}}=\mathrm {Id}$, a simple proof of the stability estimate $\Vert u\Vert _V \le C\Vert f\Vert _{V'}$, obtained for the unique solution of the operator equation $Au=f$ when *A* is injective is given in [32, Thm. 3.15]. A proof of the general case can be deduced from [22]. T-coercivity theory is a reformulation of the Banach-Nec̆as-Babus̆ka theory. The former relies on the construction of explicit inf-sup operators at the discrete and continuous levels, whereas the later develops on an abstract inf-sup condition [14].

In deriving the following results, it will be convenient to denote $\vec {{\mathbf {U}}} := ({\mathbf {U}},\,\,P)^{\top }\in {\mathbf {H}}\left( \mathbf {curl},\Omega _s\right) \times H^1(\Omega )$ and $\vec {{\mathbf {p}}} := ({\mathbf {p}},\,\,q)^{\top }\in {\mathcal {H}}_N$. We indicate with a hat equality up to a compact perturbation (e.g. $\hat{=}$).

### Space Isomorphisms

In this section, we take up the challenge of finding a suitable isomorphism ${\mathbb {X}}$. We build it separately for the function spaces in $\Omega _s$ and on the boundary $\Gamma$. Crucial hints are offered by the construction of the sign-flip isomorphism for the classical electric wave equation in [11].

We start with devising an isomorphism $\Xi$ of the volume function spaces and show that the upper-left diagonal block of ${\mathbb {G}}_{\kappa }$ satisfy a generalized Gårding inequality.

Under the assumption that the first Betti number of $\Omega _s$ is zero, there exists a bijective “scalar potential lifting” ${\mathsf {S}}:{\mathbf {N}}(\mathbf {curl},\Omega _s)\rightarrow H^1_*\left( \Omega _s\right)$ satisfying $\nabla {\mathsf {S}}\left( {\mathbf {U}}\right) ={\mathbf {U}}$. The Poincaré-Friedrichs inequality guarantees that this map is continuous.

Notice that since it also follows from the Poincaré-Friedrichs inequality that $\nabla :H_*^1(\Omega _s)\rightarrow {\mathbf {N}}(\mathbf {curl},\Omega _s)$ is injective, ${\mathsf {S}}\circ \nabla :H^1(\Omega _s)\rightarrow H_*^1(\Omega _s)$ is a bounded projection onto the space of Lebesgue measurable functions having zero mean. Its nullspace consists of the constant functions in $\Omega _s$.

#### Proposition 5.2

For any $\theta >0$ and $\beta >0$, the bounded linear operator $\Xi :{\mathbf {H}}\left( \mathbf {curl}, \Omega _s\right) \times H^1(\Omega _s)\rightarrow {\mathbf {H}}\left( \mathbf {curl}, \Omega _s\right) \times H^1(\Omega _s)$ defined byhas a continuous inverse. In other words, $\Xi$ is an isomorphism of Banach spaces.

#### Proof

By showing that $\Xi$ is a bijection, the theorem follows as a consequence of the bounded inverse theorem.

Let $\left( {\mathbf {V}}\,\,Q\right) ^{\top }\in {\mathbf {H}}\left( \mathbf {curl}, \Omega _s\right) \times H^1(\Omega _s)$. Since $\nabla Q\in {\mathbf {N}}\left( \mathbf {curl},\Omega _s\right)$, we immediately have ${\mathsf {Z}}\left( {\mathbf {V}}^{\perp }-\theta ^{-1}\nabla Q\right) ={\mathbf {V}}^{\perp }$ and $\left( \mathrm {Id}-{\mathsf {Z}}\right) \left( {\mathbf {V}}^{\perp }-\theta ^{-1}\nabla Q\right) = -\theta ^{-1}\nabla Q$. Hence, relying on the resulting observation that

$$\begin{aligned} \nabla {\mathsf {S}}\left( \left( {\mathbf {V}}^{\perp }-\theta ^{-1}\nabla Q\right) ^0\right) = -\theta ^{-1}\nabla Q \end{aligned}$$

and exploiting that $\mathbf {mean}\left( H^1_*(\Omega _s)\right) =\{0\}$, we have37Since $H^1(\Omega _s)$ decomposes into the stable direct sum of $H^1_*(\Omega _s)$ and the space of constant functions in $\Omega _s$, (37) shows that $\Xi$ is surjective.

Now, suppose that $\Xi \left( \vec {{\mathbf {V}}}\right) =\Xi \left( \vec {{\mathbf {U}}}\right)$. Then, we have

$$\begin{aligned} {\mathbf {U}}^0-{\mathbf {V}}^0 = \nabla {\mathsf {S}}\left( {\mathbf {U}}^0-{\mathbf {V}}^0\right) = \beta \,\nabla \left( \mathbf {mean}\left( Q-P\right) \right) = 0. \end{aligned}$$

Since the considerations of Sect. 4 readily yield that ${\mathbf {V}}^\perp = {\mathbf {U}}^\perp$, we conclude that ${\mathbf {V}}={\mathbf {U}}$. In turn, it follows that $\nabla P= \nabla Q$ and $\mathbf {mean}(P)=\mathbf {mean}(Q)$. Therefore, $\Xi$ is injective. $\square$

We now turn to the design of an isomorphism for the Neumann trace space ${\mathcal {H}}_N$ and prove that the lower-right block ${\mathbb {A}}_{\kappa }^{ND}$ of ${\mathbb {G}}_{\kappa }$ satisfies a generalized Gårding inequality.

#### Proposition 5.3

For any $\tau > 0$ and $\lambda >0$, the bounded linear operator $\Xi ^{\Gamma }:{\mathcal {H}}_N\rightarrow {\mathcal {H}}_N$ defined byhas a continuous inverse. In other words, $\Xi ^{\Gamma }$ is an isomorphism of Banach spaces.

#### Proof

We proceed as in proposition 5.2. Since $\left( \text {div}_{\Gamma }\right) ^{\dag }{\mathsf {Q}}_*q\in {\mathbf {X}}(\text {div}_{\Gamma },\Gamma )$, we have ${\mathsf {Z}}^{\Gamma }\left( \Xi _1^{\Gamma }(\vec {{\mathbf {p}}})\right) ={\mathbf {p}}^{\perp }-\left( \text {div}_{\Gamma }\right) ^{\dag }{\mathsf {Q}}_*q$. Using that $\mathbf {mean}\circ \text {div}_{\Gamma }=0$ and $\left( \text {div}_{\Gamma }\right) ^{\dag }\text {div}_{\Gamma }{\mathbf {p}}={\mathbf {p}}^{\perp }$, we evaluateThis shows that $\Xi ^{\Gamma }$ is surjective.

Suppose that $X^{\Gamma }(\vec {{\mathbf {p}}})=X^{\Gamma }(\vec {{\mathbf {a}}})$. It is immediate that ${\mathbf {p}}^0={\mathbf {a}}^0$. On the one hand, we obtain from $X_1^{\Gamma }(\vec {{\mathbf {p}}})=X_1^{\Gamma }(\vec {{\mathbf {a}}})$ that

38 $$\begin{aligned} {\mathbf {p}}^{\perp }-{\mathbf {a}}^{\perp } = \lambda \left( \text {div}_{\Gamma }\right) ^{\dag }\left( {\mathsf {Q}}_*q -{\mathsf {Q}}_*b\right) . \end{aligned}$$

On the other hand, $X_2^{\Gamma }(\vec {{\mathbf {p}}})=X_2^{\Gamma }(\vec {{\mathbf {a}}})$ implies that

39 $$\begin{aligned} \text {div}_{\Gamma }\left( {\mathbf {p}}-{\mathbf {a}}\right) = \lambda \,\mathbf {mean}\left( q-b\right) . \end{aligned}$$

Relying on the fact that $\text {div}_{\Gamma } = \text {div}_{\Gamma }\circ {\mathsf {Z}}^{\Gamma }$ again, combining (38) and (39) yields

$$\begin{aligned} {\mathsf {Q}}_*q + \mathbf {mean}(q) = {\mathsf {Q}}_*b + \mathbf {mean}(b). \end{aligned}$$

Evidently, (38) then also guarantees that ${\mathbf {p}}^{\perp }={\mathbf {a}}^{\perp }$. We can finally conclude that $X^{\Gamma }$ is injective and thus the result follows from the bounded inverse theorem. $\square$

In the following, we will write $\Xi ^{\Gamma }_1$ and $\Xi ^{\Gamma }_2$ for the components of the isomorphism of the trace space.

### Main Result

The main result of this work, stated in Theorem 5.6, asserts that the operator ${\mathbb {G}}_{\kappa }$ associated with the coupled system (30) is well-posed when $\kappa ^2$ lies outside the discrete set of forbidden frequencies described in [15]. It relies on two propositions, whose proofs are postponed until the end of Sect. 5.

The first claims that the *block diagonal of* ${\mathbb {G}}_{\kappa }$ (as a sum of block operators) *is T-coercive*.

#### Proposition 5.4

For any frequency $\omega \ge 0$, there exist a compact operator ${\mathsf {K}}:{\mathbf {H}}\left( \mathbf {curl}, \Omega _s\right) \times H^1(\Omega _s)\times {\mathcal {H}}_N\rightarrow {\mathbf {H}}\left( \mathbf {curl}, \Omega _s\right) \times H^1(\Omega _s)\times {\mathcal {H}}_N$, a positive constant $C>0$ and parameters $\theta >0$ and $\tau >0$, possibly depending on $\Omega _s$, $\epsilon$, $\mu$, $\kappa$ and $\omega$, such thatfor all $\vec {{\mathbf {U}}}:=\left( {\mathbf {U}}\,\,P\right) ^{\top }\in {\mathbf {H}}\left( \mathbf {curl}, \Omega _s\right) \times H^1(\Omega _s)$ and $\vec {{\mathbf {p}}}\in {\mathcal {H}}_N$.

The proof of this proposition will rely on several steps: Lemmas 5.8, 5.9 and 5.10.

The second proposition states that the off-diagonal blocks are compact operators. The proof of that fact relies on definitions and results that belong to the next technical section. It will materialize as the last piece of the puzzle that completes the proof of the T-coercivity of ${\mathbb {G}}_{\kappa }$.

#### Proposition 5.5

For any frequency $\omega \ge 0$, there exists, for a suitable choice of $\tau$, $\beta$, $\theta$ and $\lambda$, a continuous compact endomorphism ${\mathsf {K}}$ of the space ${\mathbf {H}}\left( \mathbf {curl}, \Omega _s\right) \times H^1(\Omega _s)\times {\mathcal {H}}_N$ such that40

The main result immediately follows from the two previous propositions.

#### Theorem 5.6

For any $\omega \ge 0$, there exists an isomorphism ${\mathbb {X}}_{\kappa }$ of the trial space ${\mathbf {H}}\left( \mathbf {curl}, \Omega _s\right) \times H^1(\Omega _s)\times {\mathcal {H}}_N$, and compact operator ${\mathbb {K}}:{\mathbf {H}}\left( \mathbf {curl}, \Omega _s\right) \times H^1(\Omega _s)\times {\mathcal {H}}_N\rightarrow \left( {\mathbf {H}}\left( \mathbf {curl}, \Omega _s\right) \times H^1(\Omega _s)\right) '\times {\mathcal {H}}_N'$ such thatfor some positive constant $C>0$.

#### Proof

The proof will amount to the validation that the choices of parameters in the previous propositions 5.4 and 5.5 are compatible. $\square$

The following corollary is immediate upon applying Theorem 5.1.

#### Corollary 5.7

The system operator ${\mathbb {G}}_k:{\mathbf {H}}\left( \mathbf {curl}, \Omega _s\right) \times H^1(\Omega _s)\times {\mathcal {H}}_N\rightarrow \left( {\mathbf {H}}\left( \mathbf {curl}, \Omega _s\right) \times H^1(\Omega _s)\right) '\times {\mathcal {H}}_N'$ associated with the variational problem (30) is Fredholm of index 0.

Injectivity, guaranteed when $\kappa ^2$ avoids resonant frequencies by corollary 3.2, yields well-posedness.

### T-Coercivity of the Diagonal Blocks

Equipped with the isomorphism $\Xi$, let us now study coercivity of the bilinear form ${\mathfrak {B}}_{\kappa }$ defined in (26) and associated to the Hodge–Helmholtz/Laplace operator.

#### Lemma 5.8

For any frequency $\omega \ge 0$ and parameter $\beta >0$, there exist a positive constant $C>0$ and a parameter $\theta >0$, possibly depending on $\Omega _s$, $\mu$, $\epsilon$ and $\omega$, and a compact bounded sesqui-linear form ${\mathfrak {K}}$ defined over ${\mathbf {H}}\left( \mathbf {curl}, \Omega _s\right) \times H^1(\Omega _s)$, such thatfor all $\vec {{\mathbf {U}}}:=\left( {\mathbf {U}},\,\,P\right) ^{\top }\in {\mathbf {H}}\left( \mathbf {curl}, \Omega _s\right) \times H^1(\Omega _s)$.

#### Proof

As $\mathbf {curl}\left( {\mathbf {U}}^0\right) =0$, $\mathbf {curl}\left( \nabla P\right) =0$, and $\nabla \circ \mathbf {mean} = 0$, we evaluateUpon application of the Cauchy-Schwartz inequality, the bounded sesqui-linear form

$$\begin{aligned} {\mathfrak {K}}\left( \vec {{\mathbf {U}}},\vec {{\mathbf {U}}}\right)&:= \left( \epsilon \nabla P, {\mathbf {U}}^{\perp }\right) _{\Omega _s}-\left( P,\theta {\mathsf {S}}\left( {\mathbf {U}}^0\right) \right) _{\Omega _s} +\theta \left( \epsilon {\mathbf {U}}^{\perp },{\mathbf {U}}^0\right) _{\Omega _s} \\&\quad -\omega ^2\left( \epsilon {\mathbf {U}}^0,{\mathbf {U}}^{\perp }\right) _{\Omega _s}-\omega ^2\left( \epsilon {\mathbf {U}}^{\perp },{\mathbf {U}}^{\perp }-{\mathbf {U}}^0+\beta \nabla P\right) _{\Omega _s}\\&\quad -\left( P,\theta \beta \,\mathbf {mean}(P)\right) _{\Omega _s} \end{aligned}$$

is shown to be compact by compactness of ${\mathsf {Z}}$ and the Rellich theorem. Using Young’s inequality twice with $\delta >0$, we estimate

$$\begin{aligned}&\mathfrak {Re}\left( {\mathfrak {B}}_{\kappa }\left( \vec {{\mathbf {U}}},\Xi \,\vec {{\mathbf {U}}}\right) -{\mathfrak {K}}\left( \vec {{\mathbf {U}}},\vec {{\mathbf {U}}}\right) \right) \\&\quad \ge \mu ^{-1}_{\text {max}}\,\Vert \mathbf {curl}\,{\mathbf {U}}^{\perp }\Vert ^2_{\Omega _s} + \left( \epsilon _{\text {min}}\left( \theta +\omega ^2\right) -\delta \,\epsilon _{\text {max}}\left( 1+\beta \omega ^2\right) \right) \Vert {\mathbf {U}}^0\Vert ^2_{\Omega _s} \\&\qquad +\mathfrak {Re}\left( \epsilon _{\text {min}}\,\beta -\frac{1}{\delta } \epsilon _{\text {max}}\,\left( 1 +\beta \omega ^2\right) \right) \Vert \nabla P\Vert ^2_{\Omega _s}. \end{aligned}$$

The operator $\mathbf {curl}:{\mathsf {Z}}\left( {\mathbf {H}}\left( \mathbf {curl},\Omega \right) \right) \rightarrow {\mathbf {L}}^2\left( \Omega _s\right)$ is a continuous injection, hence since its image is closed in ${\mathbf {L}}^2\left( \Omega _s\right)$, it is also bounded below. Hence, for any $\beta >0$, choose $\delta >0$ large enough, then $\theta >0$ accordingly large, and the desired inequality follows. $\square$

The complex inner products

$$\begin{aligned} \left( a,b\right) _{-1/2}&:=\int _{\Gamma }\int _{\Gamma }G_0\left( {\mathbf {x}}-{\mathbf {y}}\right) a({\mathbf {x}})\,\overline{b({\mathbf {y}})}\hbox {d}\sigma ({\mathbf {x}})\hbox {d}\sigma ({\mathbf {y}}),\\ \left( {\mathbf {a}},{\mathbf {b}}\right) _{-1/2}&:=\int _{\Gamma }\int _{\Gamma }G_0\left( {\mathbf {x}}-{\mathbf {y}}\right) {\mathbf {a}}({\mathbf {x}})\cdot \overline{{\mathbf {b}}({\mathbf {y}})}\hbox {d}\sigma ({\mathbf {x}})\hbox {d}\sigma ({\mathbf {y}}), \end{aligned}$$

defined over $H^{-1/2}(\Gamma )$ and ${\mathbf {H}}^{-1/2}\left( \text {div}_{\Gamma },\Gamma \right)$ respectively, are positive definite Hermitian forms and they induce equivalent norms on the trace spaces [9, Sec. 4.1]. Combined with the stability of the decomposition introduced in Sect. 4, this observation also allows us to conclude that

$$\begin{aligned} {\mathbf {a}}\mapsto \Vert \text {div}_{\Gamma }\left( {\mathbf {a}}\right) \Vert _{-1/2} + \Vert (\mathrm {Id}- P^{\Gamma })\,{\mathbf {a}}\Vert _{-1/2} \end{aligned}$$

also defines an equivalent norm in ${\mathbf {H}}^{-1/2}\left( \text {div}_{\Gamma },\Gamma \right)$.

Let us denote the two components of the isomorphism $\Xi$ by

$$\begin{aligned} \Xi _1(\vec {{\mathbf {U}}}):={\mathbf {U}}^{\perp }-{\mathbf {U}}^0+\nabla P,\quad \Xi _2(\vec {{\mathbf {U}}}):=-\theta \left( {\mathsf {S}}\left( {\mathbf {U}}^0\right) +\,\mathbf {mean}\left( P\right) \right) . \end{aligned}$$

We now derive an estimate similar to the one found in Lemma 5.8 that completes the proof of the coercivity of the upper-left diagonal block of ${\mathbb {G}}_{\kappa }$.

#### Lemma 5.9

For any frequency $\omega \ge 0$ and parameter $\beta >0$, there exist a positive constant $C>0$ and a parameter $\theta >0$, possibly depending on $\Omega _s$, $\mu$, $\epsilon$ and $\kappa$, and a compact linear operator ${\mathcal {K}}:{\mathbf {H}}\left( \mathbf {curl}, \Omega _s\right) \times H^1(\Omega _s)\rightarrow {\mathbf {H}}\left( \mathbf {curl}, \Omega _s\right) \times H^1(\Omega _s)$ such thatfor all $\vec {{\mathbf {U}}}:=\left( {\mathbf {U}}\,\,P\right) ^{\top }\in {\mathbf {H}}\left( \mathbf {curl}, \Omega _s\right) \times H^1(\Omega _s)$.

#### Proof

The jump condition (15a) yield $\{{\mathcal {T}}_{N}\}\cdot \mathcal {DL}_{\kappa }={\mathcal {T}}_{N}\cdot \mathcal {DL}_{\kappa }$. We deduce from [15, Sec. 6.4] that,41We consider each component of the isomorphim $\Xi$ in turn. Since ${\mathsf {Z}}\left( {\mathbf {U}}\right) \in {\mathbf {H}}^1(\Omega _s)$ [1, Lem. 3.5] and $\gamma _t{\mathbf {H}}^1(\Omega _s)$ is compactly embedded in ${\mathbf {L}}^2_t(\Gamma )$ [23, Lem. 3.2], the continous mapping $\gamma _{\tau }\circ {\mathsf {Z}}:{\mathbf {H}}\left( \mathbf {curl},\Omega _s\right) \rightarrow {\mathbf {H}}_R^{1/2}\left( \Omega _s\right)$ is compact. Therefore,

42 $$\begin{aligned} \begin{aligned} \gamma ^-_{\tau }\Xi _1\left( \vec {{\mathbf {U}}}\right)&= \gamma ^-_\tau {\mathbf {U}}^{\perp }-\gamma ^-_\tau {\mathbf {U}}^0+\beta \gamma ^-_{\tau }\nabla P\\&\,\,\,\hat{=}\, {\mathsf {Z}}^{\Gamma }\left( \gamma ^-_{\tau }{\mathbf {U}}\right) - \left( \mathrm {Id}-{\mathsf {Z}}^{\Gamma }\right) \gamma ^-_{\tau }{\mathbf {U}}+\beta \,\mathbf {curl}_{\Gamma }\left( \gamma ^-{P}\right) . \end{aligned} \end{aligned}$$

Let’s introduce expression (42) in the various terms of (41) involving $\Xi _1(\vec {{\mathbf {U}}})$. We find that

$$\begin{aligned}&\Big (\text {div}_{\Gamma }\left( \gamma _{\tau }^-{\mathbf {U}})\right) ,\text {div}_{\Gamma }\left( \gamma ^{-}_{\tau }\Xi _1\vec {{\mathbf {U}}})\right) \Big )_{-1/2}\\&\quad \hat{=} \left( \text {div}_{\Gamma }\left( \gamma _{\tau }{\mathbf {U}}\right) ,\text {div}_{\Gamma }\left( {\mathsf {Z}}^{\Gamma }\left( \gamma ^-_{\tau }{\mathbf {U}}\right) \right) \right) _{-1/2}\\&\qquad -\left( \text {div}_{\Gamma }\left( \gamma _{\tau }{\mathbf {U}}\right) ,\text {div}_{\Gamma }\left( \left( \mathrm {Id}-{\mathsf {Z}}^{\Gamma }\right) \gamma ^-_{\tau }{\mathbf {U}}\right) \right) _{-1/2}\\&\qquad +\beta \left( \text {div}_{\Gamma }\left( \gamma _{\tau }{\mathbf {U}}\right) ,\text {div}_{\Gamma }\left( \mathbf {curl}_{\Gamma }\left( \gamma ^-{P}\right) \right) \right) _{-1/2}\\&\quad = \left( \text {div}_{\Gamma }\left( \gamma ^-_{\tau }{\mathbf {U}}\right) ,\text {div}_{\Gamma }\left( \gamma ^-_{\tau }{\mathbf {U}}\right) \right) _{-1/2}. \end{aligned}$$

Similarly,

$$\begin{aligned}&- \kappa ^2\left( \gamma _{\tau }^-{\mathbf {U}},\gamma ^{-}_{\tau }\Xi _1\vec {{\mathbf {U}}}\right) _{-1/2} \hat{=} \, \,\kappa ^2\left( \left( \mathrm {Id}-{\mathsf {Z}}^{\Gamma }\right) \gamma _{\tau }^-{\mathbf {U}},\left( \mathrm {Id}-{\mathsf {Z}}^{\Gamma }\right) \gamma ^-_{\tau }{\mathbf {U}}\right) _{-1/2}\\&-\beta \kappa ^2\left( \left( \mathrm {Id}-{\mathsf {Z}}^{\Gamma }\right) \gamma _{\tau }^-{\mathbf {U}},\mathbf {curl}_{\Gamma }\left( \gamma ^-{P}\right) \right) _{-1/2} \end{aligned}$$

and

$$\begin{aligned} \left( \gamma ^{-}_{\tau }\Xi _1\vec {{\mathbf {U}}},\mathbf {curl}_{\Gamma }\left( \gamma ^-\left( P\right) \right) \right) _{-1/2}&\hat{=}&-\left( \left( \mathrm {Id}-{\mathsf {Z}}^{\Gamma }\right) \gamma ^-_{\tau }{\mathbf {U}},\mathbf {curl}_{\Gamma }\left( \gamma ^-\left( P\right) \right) \right) _{-1/2} \\&\quad +\beta \left( \mathbf {curl}_{\Gamma }\left( \gamma ^-{P}\right) ,\mathbf {curl}_{\Gamma }\left( \gamma ^-\left( P\right) \right) \right) _{-1/2}. \end{aligned}$$

We now want to evaluate the terms involving $\Xi _2(\vec {{\mathbf {U}}})$. We introduce

$$\begin{aligned} \mathbf {curl}_{\Gamma }\left( \gamma ^{-} \Xi _2\vec {{\mathbf {U}}}\right) =-\theta \gamma ^-_{\tau }\nabla \left( {\mathsf {S}}({\mathbf {U}}^0)+\mathbf {mean}(P)\right) =-\theta \left( \mathrm {Id}-{\mathsf {Z}}^{\Gamma }\right) \gamma ^-_{\tau }{\mathbf {U}}, \end{aligned}$$

in (41) to obtain

$$\begin{aligned} -\left( \gamma _{\tau }^-{\mathbf {U}},\mathbf {curl}_{\Gamma }\left( \gamma ^{-} \Xi _2\vec {{\mathbf {U}}}\right) \right) _{-1/2} =\theta \left( \left( \mathrm {Id}-{\mathsf {Z}}^{\Gamma }\right) \gamma ^-_{\tau }{\mathbf {U}},\left( \mathrm {Id}- {\mathsf {Z}}^{\Gamma }\right) \gamma _{\tau }{\mathbf {U}}\right) _{-1/2} \end{aligned}$$

Using Young’s inequality twice with $\delta >0$,The operator $\mathbf {curl}_{\Gamma }:H^1_*(\Omega _s)\rightarrow {\mathbf {H}}^{-1/2}\left( \text {div}_{\Gamma },\Gamma \right)$ is a continuous injection [15, Lem. 6.4]. It is thus bounded below. Since the mean operator has finite rank, it is compact. Therefore, for any $\beta >0$, choose $\delta >0$ large enough, then $\theta >0$ accordingly large, and the desired inequality follows by equivalence of norms. $\square$

In the next lemma, we prove coercivity of the lower diagonal block of the coupling operator ${\mathbb {G}}_{\kappa }$.

#### Lemma 5.10

For any frequency $\omega \ge 0$, there exist a compact linear operator ${\mathcal {K}}:{\mathcal {H}}_N\rightarrow {\mathcal {H}}_D$, a positive constants $C>0$ and parameters $\tau >0$ and $\lambda >0$, possibly depending on $\Omega _s$, $\mu$, $\epsilon$ and $\kappa$, such thatfor all $\vec {{\mathbf {p}}}\in {\mathcal {H}}_N$. In particular, for $\mathfrak {Re}\left( k^2\right) \ne 0$, the inequality holds with $\tau = 1/\kappa ^2$.

#### Proof

The jump conditions (15b) yield $\{{\mathcal {T}}_D\}\cdot \mathcal {SL}\left( \vec {{\mathbf {p}}}\right) ={\mathcal {T}}_D\cdot \mathcal {SL}\left( \vec {{\mathbf {p}}}\right)$. We deduce from [15, Sec. 6.3] and the compact embedding of ${\mathbf {X}}\left( \text {div}_{\Gamma },\Gamma \right)$ into ${\mathbf {H}}_R^{-1/2}(\Gamma )$ that

$$\begin{aligned} \Big \langle {\mathcal {T}}_D\cdot \mathcal {SL}\left( \vec {{\mathbf {p}}}\right) ,\Xi ^{\Gamma }\vec {{\mathbf {p}}}\Big \rangle&\hat{=}-\left( {\mathbf {p}}^0,\Xi ^{\Gamma }_1({\mathbf {p}})\right) _{-1/2} -\left( q,\text {div}_{\Gamma }\left( \Xi ^{\Gamma }_1({\mathbf {p}})\right) \right) _{-1/2} \\&\quad -\left( \text {div}_{\Gamma }({\mathbf {p}}),\Xi ^{\Gamma }_2\vec {{\mathbf {p}}}\right) _{-1/2} -\kappa ^2\left( q,\Xi ^{\Gamma }_2\left( \vec {{\mathbf {p}}}\right) \right) _{-1/2}\\&\hat{=}\left( {\mathbf {p}}^0,{\mathbf {p}}^0\right) _{-1/2} -\left( q,\text {div}_{\Gamma }({\mathbf {p}}^{\perp })\right) _{-1/2} +\lambda \left( q,{\mathsf {Q}}_*q\right) _{-1/2} \\&\quad +\tau \left( \text {div}_{\Gamma }({\mathbf {p}}),\text {div}_{\Gamma }({\mathbf {p}})\right) _{-1/2} + \tau \kappa ^2\left( q,\text {div}_{\Gamma }({\mathbf {p}}^{\perp })\right) _{-1/2}. \end{aligned}$$

When $\mathfrak {Re}\left( \kappa ^2\right) > 0$, setting $\tau =1/\kappa ^2$ immediately yields the existence of a compact linear operator ${\mathcal {K}}:{\mathcal {H}}_N\rightarrow {\mathcal {H}}_D$ such that

$$\begin{aligned}&\Big \langle {\mathcal {T}}_D\cdot \mathcal {SL}\left( \vec {{\mathbf {p}}}\right) ,\Xi ^{\Gamma }\vec {{\mathbf {p}}}\Big \rangle + \Big \langle {\mathcal {K}}\vec {{\mathbf {p}}},\Xi ^{\Gamma }\vec {{\mathbf {p}}}\Big \rangle \\&\quad \ge C\left( \Vert \text {div}_{\Gamma }\left( {\mathbf {p}}\right) \Vert ^2_{-1/2} + \Vert {\mathbf {p}}^0\Vert ^2_{-1/2} + \Vert {\mathsf {Q}}_*q\Vert ^2_{-1/2}\right) . \end{aligned}$$

When $\kappa ^2 = 0$, the same inequality is obtained for any $\lambda >0$ by using Young’s inequality as in the proof of Lemma 5.9 and choosing $\tau$ large enough. The claimed inequality follows by equivalence of norms. $\square$

Equipped with the previous three lemmas, we are now ready to prove Proposition 5.4.

#### Proof of Proposition 5.4

For any parameters $\beta >0$ and $\lambda >0$, the choices of $\delta$ and $\theta$ in the proofs of Lemma 5.8 and Lemma 5.9 are not mutually exclusive. The choice of $\tau$ in Lemma 5.10 is independent of the choice of $\theta$.    $\square$

### Compactness of the Off-Diagonal Blocks

Finally, The off-diagonal blocks remain to be considered. We will show that, up to compact perturbations, a suitable choice of parameters in the isomorphisms $\Xi$ and $\Xi ^{\Gamma }$ of the test space leads to a skew-symmetric pattern in ${\mathbb {G}}_{\kappa }$. In other words, up to compact terms, the volume and boundary parts of the system decouples over the space decompositions introduced in Sect. 4.

#### Proof of Proposition 5.5

The isomorphisms $\Xi$ and $\Xi ^{\Gamma }$ were designed so that favorable cancellations occur in evaluating the left hand side of (40).

From the jump properties (15b), we have $\{{\mathcal {T}}_N\}\mathcal {SL}_{\kappa } = {\mathcal {T}}_N^-\mathcal {SL}_{\kappa } -(1/2)\mathrm {Id}$. Therefore, as in (24), we evaluate43where we have used that the finite rank of the mean operator implies compactness.

Similarly, using Proposition 2.3, we find4445Many terms in these equations can be combined and asserted compact by (35a) and (35b). They are indicated in . When summing the real parts of (43) and (45), the terms in  cancel. Relying on (12a) to (12d), some terms amount to compact perturbations so that we may replace $\kappa$ and ${\tilde{\kappa }}$ by zero in those instances. We have arrived at the following identity:We claim that the terms colored in  are compact. Indeed, the integral identities of Sect. 2.1 together with equality (11) yield

$$\begin{aligned}&\langle \gamma ^-_n\varvec{\Psi }_0\left( {\mathbf {p}}\right) ,\gamma ^- {\mathsf {S}}\left( \overline{{\mathbf {U}}}^0\right) \rangle _{\Gamma }\nonumber \\&\quad \le \left( \Vert \psi _0\left( \text {div}_{\Gamma }{\mathbf {p}}\right) \Vert _{L^2(\Omega _s)}+\Vert \varvec{\Psi }_0\left( {\mathbf {p}}\right) \Vert _{{\mathbf {L}}^2(\Omega _s)}\right) \Vert \overline{{\mathbf {U}}}^0\Vert _{{\mathbf {L}}^2(\Omega _s)},\\&\langle \gamma _n^-\varvec{\Psi }_0\left( \overline{{\mathbf {p}}}\right) ,\gamma ^-P\rangle _{\Gamma }\nonumber \\&\quad \le \left( \Vert \psi _0\left( \text {div}_{\Gamma }\overline{{\mathbf {p}}}\right) \Vert _{L^2(\Omega _s)}+\Vert \varvec{\Psi }_0\left( \overline{{\mathbf {p}}}\right) \Vert _{{\mathbf {L}}^2(\Omega _s)}\right) \Vert P\Vert _{H^1(\Omega _s)}\\&\langle \gamma ^-_n\varvec{\Psi }_0\left( \left( \text {div}\right) ^{\dag }Q_*{\overline{q}}\right) ,\gamma ^-P\rangle _{\Gamma },\nonumber \\&\quad \le \left( \Vert \psi _0\left( Q_*q\right) \Vert _{L^2(\Omega _s)}+\Vert \varvec{\Psi }_0\left( \text {div}_{\Gamma }\overline{{\mathbf {p}}}\right) \Vert _{{\mathbf {L}}^2(\Omega _s)}\right) \Vert P\Vert _{H^1(\Omega _s)}. \end{aligned}$$

Since $\psi _{0}:H^{-1/2}(\Gamma )\rightarrow H^1(\Omega _s)$ and $\varvec{\Psi }_{0}:{\mathbf {H}}^{-1/2}(\Gamma )\rightarrow {\mathbf {H}}^1(\Omega _s)$ are continuous, compactness is guaranteed by Rellich’s Theorem.

To go further, we need to settle for a choice of parameters in the volume and boundary isomorphisms. Choose $\tau$ to satisfy the requirements of Lemma 5.10, then set $\beta = \tau$. We are still free to let $\theta$ satisfy both Lemmas 5.8 and 5.9, and then choose $\lambda = \theta$.

Under this choice of parameters, the terms in  vanish, because we have $\langle {\mathbf {p}}^{\perp },\gamma _t^-\nabla {\overline{P}}\rangle _{\tau } = \langle {\mathbf {p}}^{\perp },\nabla _{\Gamma }\gamma ^-{\overline{P}}\rangle _{\tau }=-\langle \text {div}_{\Gamma }\left( {\mathbf {p}}^{\perp }\right) ,\gamma ^-{\overline{P}}\rangle _{\Gamma }$, and similarly

$$\begin{aligned} \langle \gamma ^-_t{\mathbf {U}}^0,\left( \text {div}_{\Gamma }\right) ^{\dag }Q_*{\overline{q}}\rangle _{\tau }&=\langle \gamma ^-_t\nabla \nonumber {\mathsf {S}}\left( {\mathbf {U}}^0\right) ,\left( \text {div}_{\Gamma }\right) ^{\dag }Q_*{\overline{q}}\rangle _{\tau }\\&=-\langle \gamma ^-{\mathsf {S}}\left( {\mathbf {U}}^0\right) ,Q_*{\overline{q}}\rangle _{\Gamma }. \end{aligned}$$

Finally, relying on (10a), (10b) and (11) once more, we observe that

$$\begin{aligned}&\langle \gamma _R^-\varvec{\Psi }_0\left( {\mathbf {p}}^{\perp }\right) ,\gamma _t^-\nabla {\overline{P}}\rangle _{\tau } =\left( \mathbf {curl}\,\mathbf {curl}\,\varvec{\Psi }_0\left( {\mathbf {p}}^{\perp }\right) ,\nabla P\right) _{\Omega _s}\\&\quad =\left( \nabla \psi _0\left( \text {div}_{\Gamma }{\mathbf {p}}^{\perp }\right) ,\nabla P\right) _{\Omega _s} = \langle \gamma ^-_n\nabla \psi _0\left( \text {div}_{\Gamma }{\mathbf {p}}^{\perp }\right) ,\gamma ^-{\overline{P}}\rangle _{\Gamma }. \end{aligned}$$

A similar derivation shows that

$$\begin{aligned} \langle \gamma ^-_n\nabla \psi _{0}\left( q\right) , \gamma ^-{\mathsf {S}}\left( \overline{{\mathbf {U}}}^0\right) \rangle _{\Gamma }\,\hat{=}\, \langle \gamma _R^-\varvec{\Psi }_{0}\left( \left( \text {div}_{\Gamma }\right) ^{\dag }Q_*{\overline{q}}\right) ,\gamma _t^-{\mathbf {U}}^0 \rangle _{\tau }. \end{aligned}$$

We conclude that for such a choice of parameters,which concludes the proof of this proposition. $\square$

## Conclusion

In Sect. 3 we have proposed a system of equations coupling the *mixed formulation* of the variational form of the Hodge–Helmholtz and Hodge–Laplace equation with *first-kind* boundary integral equations. Well-posedness of the coupled problem was obtained using a T-coercivity argument demonstrating that the operator associated to the coupled variational problem was Fredholm of index 0. When $\kappa ^2\in {\mathbb {C}}$ avoids resonant frequencies, the operator’s injectivity is guaranteed, and thus stability of the problem is obtained along with the existence and uniqueness of the solution. For such $\kappa ^2$, Proposition 3.1 shows how solutions to the coupled variational problem are in one-to-one correspondence with solutions of the transmission system. In principle, the CFIE-type stabilization strategy applicable to transmission problems for the scalar Helmholtz operator [24] or the electric wave equation [25] could also be attempted here to get rid of the spurious resonances haunting the coupled problem (30), but such developments lie outside the scope of this work.

The symmetrically coupled system (30) offers a variational formulation of the transmission problem (3) in well-known energy spaces suited for discretization by finite and boundary elements. It is therefore a promising starting point for Galerkin discretization.
